# Mussel‐inspired self‐assembly of silver nanoclusters into multifunctional silver aerogels for enhanced catalytic and bactericidal applications

**DOI:** 10.1002/EXP.20240034

**Published:** 2024-06-26

**Authors:** Yunshan Gao, Jie Xu, Shaohua Qu, Yixiao Li, Gleb B. Sukhorukov, Li Shang

**Affiliations:** ^1^ State Key Laboratory of Solidification Processing School of Materials Science and Engineering Northwestern Polytechnical University Xi'an China; ^2^ A.V. Zelmann Center for Neurobiology and Brain Rehabilitation Skolkovo Institute of Science and Technology Moscow Russia

**Keywords:** aerogels, antibacterial activity, dopamine, dye degradation, silver nanoclusters

## Abstract

Silver nanoclusters (AgNCs) have shown broad application prospects in catalysis, sensing, and biological fields. However, the limited stability of AgNCs has become the main challenge restricting their practical application in complex environments. Herein, a mussel‐inspired, dopamine‐assisted self‐assembly approach is reported to fabricate 3D AgNC aerogels (PDA/AgNCs), which possess significantly enhanced structural stability and synergistic functional properties. The prepared AgNC aerogels display a hierarchical network structure with an ultrafine ligament size of 10.3 ± 1.2 nm and a high specific surface area of 50.7 m^2^ g^−1^. The gelation mechanism is elucidated by in‐depth characterization and time‐lapse monitoring of the gelation process vis spectroscopic and microscopic approaches. Owing to the distinct features of aerogels and the synergistic effect of AgNCs and PDA, the fabricated aerogels can not only efficiently decolorize dyes with a faster kinetic than individual AgNCs, but also exhibit remarkable broad‐spectrum antimicrobial activity. Consequently, a conceptual water‐treatment device is established by depositing PDA/AgNC aerogels on the cotton substrate, which shows good performance in both catalytic dye degradation and bacterial killing in the flowing system. This mussel‐inspired self‐assembly strategy has great potential in developing robust AgNC‐based functional materials, which also provides a new guideline for designing sophisticated materials with integrated functions and synergistic properties.

## Graphical abstract

A mussel‐inspired self‐assembly strategy is proposed to fabricate silver aerogels based on ultrasmall silver nanoclusters through a dopamine‐assisted gelation process. The fabricated silver aerogels possess excellent catalytic and antibacterial properties owing to the synergistic effect of silver nanoclusters and polydopamine, which holds promising potential as novel multifunctional materials for application in environmental and healthcare fields.

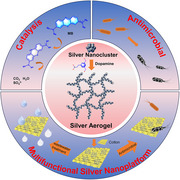

## 1 INTRODUCTION

Metal nanoclusters (NCs), which are composed of several to hundreds of metal atoms, exhibit many characteristics that are significantly different from large metal nanoparticles due to their ultrasmall size.^[^ ^1^ ^]^ Consequently, metal NCs have shown great potential in the fields of biosensors,^[^ ^2^ ^]^ fluorescence imaging,^[^ ^3^ ^]^ optical devices,^[^ ^4^ ^]^ disease treatment,^[^ ^5^ ^]^ and chemical catalysis.^[^ ^6^ ^]^ Among various metal NCs reported so far, silver nanoclusters (AgNCs) have attracted intensive attention due to their distinct physicochemical properties, such as tunable fluorescence, excellent antibacterial activity, and unique catalytic features.^[^ ^7^ ^]^ However, AgNCs typically possess limited stability because of their high reactivity and easy oxidation in the ambient environment, which largely restricts their further utility in many practical applications. Therefore, enhancing the stability of AgNCs has been considered one of the most important challenges in the development of high‐performance Ag‐based nanomaterials for versatile advanced applications.

Indeed, various enhancement strategies have been developed in the past decades, such as ligand engineering,^[^ ^8^ ^]^ metal doping^[^ ^9^ ^]^ and self‐assembly,^[^ ^10^ ^]^ in order to endow AgNCs with improved photophysical properties and chemical stability. In particular, as an emerging type of non‐covalent strategy, self‐assembly can not only inherit the property of the functional unit but also bring new properties from the assembly and easily regulate its performance by controlling the structure of the assembly. For example, Feng et al.^[^ ^10^ ^]^ recently introduced positively‐charged choline chloride to modulate the structure of the AgNC assembly, providing a simple method to optimize their optical properties and chirality by adjusting the lateral interaction between adjacent nanofibers. In another study, AgNCs were complexed with tartaric acid through hydrogen bonding, forming chiral cocrystals with distinct circularly polarized phosphorescence properties.^[^ ^11^ ^]^ Alternatively, Liu et al.^[^ ^12^ ^]^ encapsulated AgNCs into phosphatidylcholine liposomes via self‐assembly to endow them with enhanced stability and better biocompatibility than single AgNC. Despite these advances, it remains challenging to fabricate robust AgNC‐based multi‐functional materials in a controllable yet efficient manner.

**SCHEME 1:**
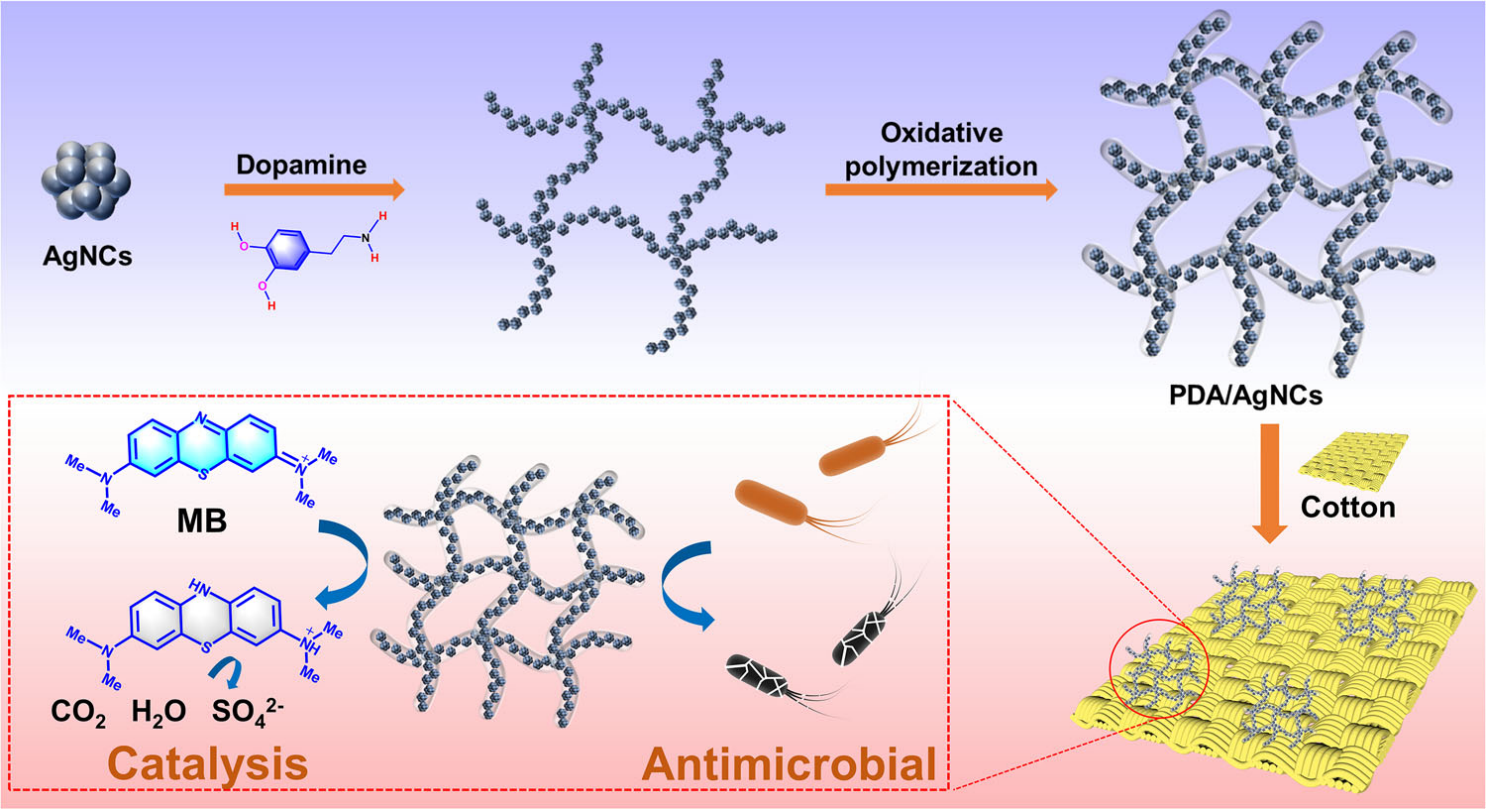
Schematic illustration of the preparation process of PDA/AgNC aerogels and their applications. [Correction added on 9th July 2024, after first online publication: Scheme 1 image was replaced with correct one.]

Recently, mussel‐inspired polyphenols have shown great potential in materials engineering owing to their good controllability, strong surface adhesion, and simplicity. For instance, dopamine can self‐polymerize on the surface of versatile materials at either microscale or nanoscale by non‐covalent/covalent interactions,^[^ ^13^ ^]^ which can act as a promising scaffold for constructing self‐assembled materials.^[^ ^14^ ^]^ Thus, we envision that this mussel‐inspired engineering strategy may provide an innovative solution to fabricate robust AgNC‐based materials, which have been not exploited yet. Herein, we report a polydopamine (PDA)‐based approach to prepare AgNC‐nucleated multifunctional metal aerogels. As illustrated in Scheme 1, our approach involves interfacial assembly, oxidation polymerization, and step‐wise gelation. The prepared PDA/AgNC aerogels possess a 3D network with ultrafine ligament size and densely‐loaded AgNCs. While abundant porous structures of PDA‐templated aerogels greatly facilitate the interfacial catalytic reaction of embedded AgNCs, the strong adhesion capability of the PDA layer as well as the antibacterial activity of AgNCs can synergistically exhibit promising bactericidal properties. Consequently, a conceptual water‐treatment device based on PDA/AgNC aerogels can be established, which showed good performances in both catalytic dye degradation and bacterial killing. Therefore, this mussel‐inspired self‐assembly strategy holds great potential in developing robust metal NC‐based multifunctional materials.

## 2 RESULTS AND DISCUSSION

### 2.1 Fabrication and characterization of PDA/AgNC aerogels

**FIGURE 1:**
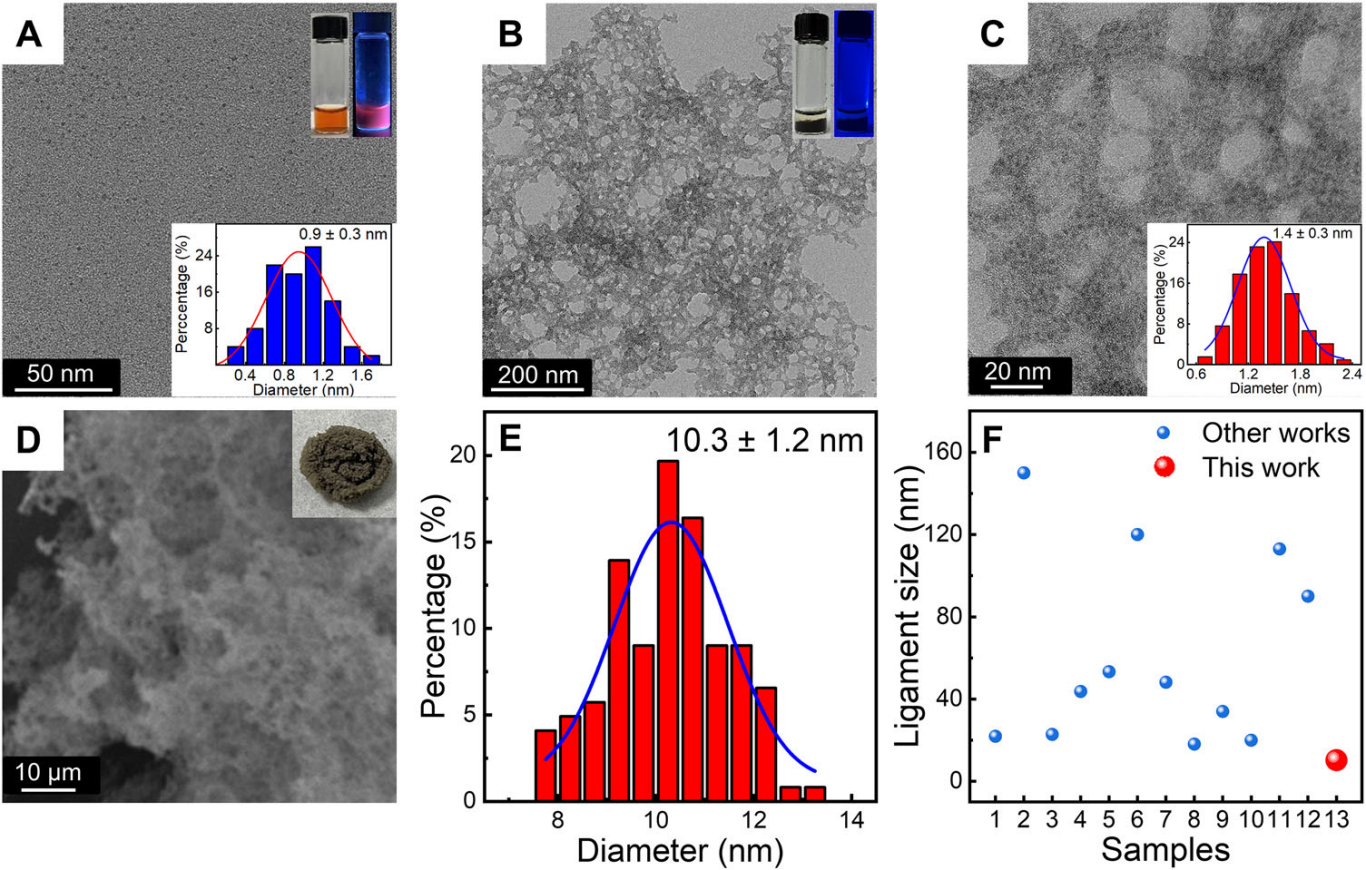
Morphological characterization of AgNCs and PDA/AgNC aerogels. (A) TEM image of DHLA‐AgNCs. Inset: photographs of AgNCs solution under the visible light (upper left) and UV light (upper right); the size histograms of DHLA‐AgNCs (bottom). (B) TEM image of PDA/AgNC aerogels. Inset: photographs of PDA/AgNC hydrogels under visible light (left) and UV light (right). (C) Representative high‐resolution TEM image of PDA/AgNC aerogels. Inset: the size histograms of individual AgNCs in the PDA/AgNC aerogels. (D) SEM image of PDA/AgNC aerogels. Inset: photograph of PDA/AgNC aerogels obtained by freeze‐drying. (E) The histogram of the ligament size of PDA/AgNC aerogels based on TEM images. (F) Summary of the ligament size of various silver aerogels reported in the literature (blue dots) and in the present work (red dot). References denoted by blue dots: Sample 1, Du et al.;^[^ ^16^ ^]^ Sample 2, Zhu et al.;^[^ ^17^ ^]^ Samples 3–5, Gao et al.;^[^ ^18^ ^]^ Sample 6, Huang et al.;^[^ ^19^ ^]^ Sample 7, Du et al.;^[^ ^20^ ^]^ Sample 8, Du et al.;^[^ ^21^ ^]^ Sample 9, Yuan et al.;^[^ ^22^ ^]^ Sample 10, Panigrahy et al.;^[^ ^23^ ^]^ Sample 11, Jung et al.;^[^ ^24^ ^]^ Sample 12, Georgi et al.^[^ ^25^ ^].^

Water‐soluble AgNCs stabilized by the short thiolate ligand (dihydrolipoic acid, DHLA) were first synthesized via a simple one‐pot strategy. The as‐prepared AgNCs possess three characteristic absorption peaks at 325 nm, 425, and 500 nm, as well as red fluorescence emission peaks at 690 nm (Figure S1), which are consistent with those reported in the literature.^[^ ^15^ ^]^ Transmission electron microscopy (TEM) images showed that these AgNCs are uniformly dispersed with an average particle size of 0.9 ± 0.3 nm (Figure 1A), further confirming the successful synthesis of ultrasmall‐sized AgNCs.

After adding DA into the solution of AgNCs at pH 10, the supernatant gradually became colorless and the grayish‐black gel gradually deposited at the bottom of the bottle. Meanwhile, the red fluorescence of AgNCs disappeared in the final product, as shown in Figure 1B, indicating the successful preparation of PDA/AgNC composite. After freeze‐drying, PDA/AgNC aerogels were obtained and characterized by TEM and scanning electron microscopy (SEM). As shown in Figure 1B–D, the PDA/AgNCs exhibit a 3D porous network structure composed of interconnected nanowires. According to the high magnification TEM image (Figure 1C), AgNCs mostly exist in the form of individual particles within the network structure, and there is a thin out‐layer with a low density, presumably formed by the oxidative polymerization of DA (also known as PDA). AgNCs in the gel network are evenly distributed with an average particle size of 1.4 ± 0.3 nm, which is larger than that of original AgNCs (0.9 ± 0.3 nm). This observation indicates that AgNCs have grown slightly during the assembly process, but the final size is still less than 2 nm. The obtained PDA/AgNC aerogels exhibit networks comprised of nanowires with a ligament size of 10.3 ± 1.2 nm (Figure 1E). We note that such a small ligament size is the smallest one among the reported Ag aerogels (Figure 1F and Table S1), which is likely attributed to the ultrasmall size of our building blocks, AgNCs. Studies have shown that the ligament size of the network structure plays a crucial role in defining their properties, and aerogels with small ligament sizes typically exhibit large specific surface area and excellent catalytic performance.^[^ ^26^ ^]^

**FIGURE 2:**
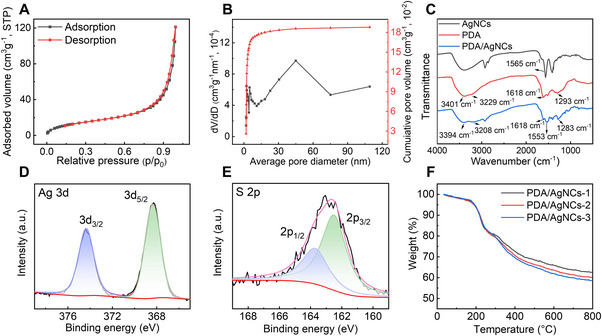
Characterization of PDA/AgNC aerogels. (A) Nitrogen adsorption‐desorption isotherm and (B) the corresponding BJH pore size distributions of PDA/AgNC aerogels. (C) FTIR spectra of AgNCs, PDA, and PDA/AgNC aerogels. High‐resolution XPS spectra of PDA/AgNCs: (D) Ag 3d, (E) S 2p. (F) TGA curves of PDA/AgNCs‐1, PDA/AgNCs‐2, and PDA/AgNCs‐3.

Subsequently, the specific surface area and porosity of PDA/AgNC aerogels were measured by N_2_ physisorption isotherm. As seen in Figure 2A, the N_2_ physisorption isotherm of the PDA/AgNC aerogels has characteristics of both type II and type IV isotherms, which indicates that mesopores and macropores widely exist in the structure of the aerogels.^[^ ^27^ ^]^ The specific surface area was calculated to be 50.7 m^2^ g^−1^ according to the Bruno–Emmett–Teller (BET) method, which is also superior to most of the Ag aerogel materials reported in the literature (Table S1). The pore size distribution indicates that micropores (<2 nm) and mesopores (2–50 nm) are widely present in PDA/AgNC aerogels (Figure 2B), which is consistent with the SEM results in Figure 1D.

The X‐ray diffraction (XRD) analysis was conducted to detect the phase composition of PDA/AgNC aerogels, as shown in Figure S2. The XRD pattern of PDA displays a broad diffraction peak, indicating the amorphous feature of PDA. Compared with PDA, there are three characteristic peaks located at 2*θ* = 38.1°, 44.2°, and 64.4° in the spectrum of PDA/AgNC aerogels, which can be assigned to the (111), (200), and (220) reflection planes of Ag, respectively. This result not only confirms the existence of Ag in the PDA/AgNCs, but also indicates that the size of AgNCs in the PDA/AgNCs increased compared to the original AgNCs.

The physicochemical properties of PDA/AgNC aerogels were first studied by Fourier transform infrared spectroscopy (FTIR, Figure 2C). The characteristic absorption peaks of the main chemical groups in both PDA and AgNCs appeared in the FTIR spectra of PDA/AgNC aerogels, suggesting the presence of both components. Compared with that of DHLA‐AgNCs, the stretching vibration absorption band of C═O bond in PDA/AgNC composites moves from 1565 cm^−1^ to 1553 cm^−1^. Meanwhile, the absorption band at 3200–3500 cm^−1^ corresponding to the tensile vibration of ─OH and N─H functional group of PDA/AgNC composites becomes broad, and the vibration absorption band of the phenol group shifts to a lower frequency, as compared with PDA. These changes also indicate the existence of hydrogen bond between phenolic hydroxyl group and amine group in PDA and oxygen atoms on the surface of DHLA‐AgNCs.^[^ ^28^ ^]^

In order to further characterize the chemical composition of PDA/AgNC aerogels, X‐ray photoelectron spectroscopy (XPS) was employed to analyze the valance state of main elements in the aerogels. As shown in Figure 2D, the binding energies of Ag 3d_5/2_ and Ag 3d_3/2_ in the XPS spectrum of PDA/AgNC aerogels are 368.3 eV and 374.3 eV, respectively. This result indicates that Ag exists in the PDA/AgNC aerogels as a metal state,^[^ ^29^ ^]^ which is consistent with the state of Ag in DHLA‐AgNCs.^[^ ^15^ ^]^ In addition, the S 2p spectrum of PDA/AgNC aerogels also shows similar features as that of AgNCs before the assembly.^[^ ^15^ ^]^ The binding energies of S 2p_3/2_ and S 2p_1/2_ doublet peaks located at 162.5 eV and 163.7 eV, respectively (Figure 2E), which can be attributed to the S atom bound to Ag. In addition, there is no typical peak of oxidized sulfur (ca. 168.0 eV), indicating rather good stability of AgNCs in the fabricated aerogels thanks to the strong protecting capability of DHLA ligands and the aerogel structures.

The concentration of the gelator is known to play an important role in the assembly process of nanoparticles in forming aerogels, which can affect their final morphology and structures.^[^ ^27^, ^30^ ^]^ Therefore, the effect of DA with different concentrations on the fabrication of PDA/AgNC aerogels was studied in detail by means of UV–Vis absorption spectroscopy and TEM. While DHLA‐AgNCs exhibit good stability in aqueous solution, they will become unstable after the pretreatment by the purification. During the purification, most of the free ligands were removed, leading to a “metastable state” of AgNCs, in which AgNCs tend to fuse with each other to form relatively large and stable forms. In the absence of dopamine, large Ag nanoparticles will form without the generation of gel‐like structures (Figure S3). In contrast, after adding a certain amount of DA (2.5–20 mm), AgNCs would undergo specific assembly, which gradually deposited at the bottom of the solution under the action of gravity. Herein, the speed of the sedimentation varied slightly depending on the concentration of DA (Figure S4). When the concentration of DA increased from 2.5 to 20 mm, the time to start the sedimentation decreased from 144 to 6 h. Obviously, reasonably increasing the concentration of DA is conducive to accelerating the gelation process of the system. However, when the concentration of DA is too low (e.g., 1 mm), the reaction solution only exhibited color changes without sedimentation.

Meanwhile, we found that the morphology and structure of PDA/AgNC assemblies will change with the concentration of DA. At a relatively low concentration of DA (e.g., 2.5 mm), the network structure of the assembly is mostly discontinuous, with the appearance of many unconnected/entangled nanowires. With the increase of DA concentration (5–20 mm), AgNCs were directionally assembled into a gel network structure (Figure S5). Meanwhile, with increasing the DA concentration, the amount of formed PDA layer on the surface of the assembly significantly increased. Owing to the strong adhesive property of the PDA layer, inter‐fusion and entanglement between nearby nanowires will be enhanced, resulting in the formation of network structures with thicker networks. Consequently, the ligament size of the gel network structure of the assembly increased at a higher amount of DA. According to Figure 1E and Figure S6, when the DA concentration is 5, 10, and 20 mm, the average ligament size of the obtained PDA/AgNC aerogels was measured to be 10.3 ± 1.2 nm (named as PDA/AgNCs‐1), 14.2 ± 3.2 nm (named as PDA/AgNCs‐2) and 20.8 ± 4.3 nm (named as PDA/AgNCs‐3), respectively. This fact is consistent with the rising up of the scattering intensity of PDA/AgNC solution within the range of 400–700 nm in the absorption spectra (Figure S7). Further semi‐quantitative analysis by XPS revealed that the Ag/N ratio in PDA/AgNCs‐1, PDA/AgNCs‐2, and PDA/AgNCs‐3 is 0.93, 0.68, and 0.36, respectively (Figure S8), indicating that the relative content of Ag in the final materials decreases with the increase of the DA concentration during the synthesis process.

In order to further evaluate the composition of as‐prepared PDA/AgNC aerogels, thermogravimetric analysis (TGA) was carried out. As shown in Figure 2F, PDA/AgNC aerogels mainly exhibit three thermal decomposition stages in the TGA curves. The first decomposition stage is 35–100°C, corresponding to the evaporation of water in the system. The second stage is presumably attributed to the decomposition of DA in the range of 100–300°C, which acts as a linker between AgNCs in the system.^[^ ^31^ ^]^ The final stage is the decomposition of PDA at temperatures above 300°C.^[^ ^28^ ^]^ Considering that the thermal stability of Ag under an argon atmosphere does not cause additional weight loss, the higher the Ag content, the greater the residual mass of the materials at 800°C. Indeed, the residual mass of PDA/AgNCs‐1, PDA/AgNCs‐2, and PDA/AgNCs‐3 at 800°C was measured to be ≈62.5 wt%, 60.0 wt%, and 58.6 wt%, respectively, which agrees well with the above XPS result.

Previous studies showed that pH also affects the polymerization process of DA,^[^ ^32^ ^]^ thus we further investigated the effect of pH on the formation of PDA/AgNC aerogels. Due to the protonation of the carboxylic groups of DHLA ligands on the surface of AgNCs at relatively low pH, aggregation and precipitation will occur in the reaction solution with pH of 6.0 and 8.5 (Figure S9). At pH 6.0, the appearance of weak absorption bands in the range of 400–550 nm indicated the formation of large‐sized Ag nanostructures (Figure S10).^[^ ^33^ ^]^ TEM images further confirmed the formation of disordered aggregates of Ag NPs without obvious gel structures (Figure S11). When the pH was increased to 10 or 12, a more complete gel structure was formed. However, the formed structures did not settle down at pH 12 likely because of the degradation of PDA in strong alkaline condition.^[^ ^34^ ^]^ Thus, pH 10 was considered as the optimal condition for the gelation of AgNCs.

### 2.2 Gelation mechanism of PDA/AgNC aerogels

**FIGURE 3:**
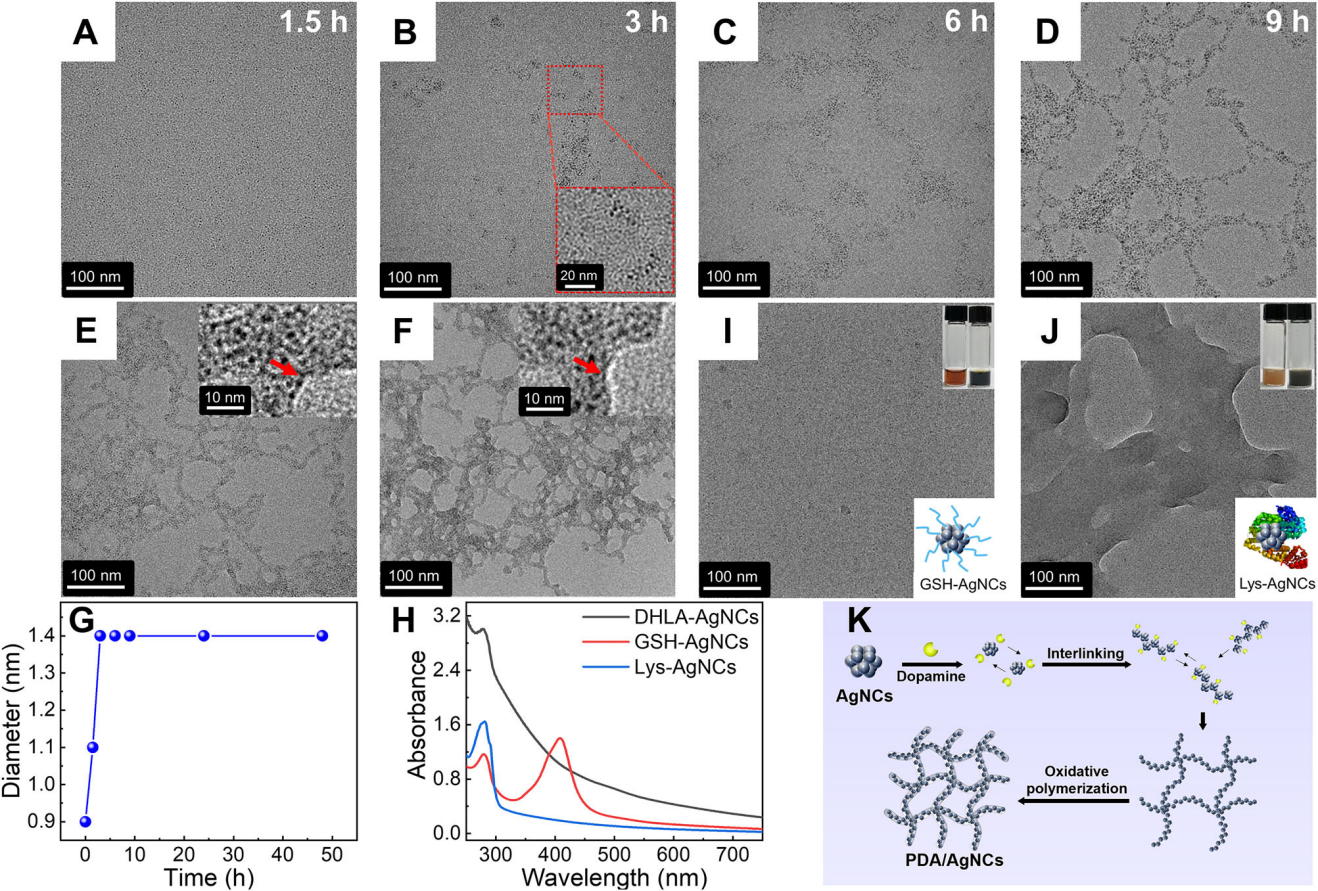
Analysis of the gelation mechanism of PDA/AgNC aerogels. TEM images for the assembly process of AgNCs at (A) 1.5 h, (B) 3 h, (C) 6 h, (D) 9 h, (E) 24 h, and (F) 48 h, upon the addition of 5 mm DA. Insets: enlarged image. (G) The size of AgNCs at different timepoints during the assembly process. (H) UV–vis absorption spectra of DHLA‐AgNCs, GSH‐AgNCs, and Lys‐AgNCs upon the addition of 5 mm DA for 48 h. TEM images of (I) GSH‐AgNCs and (J) Lys‐AgNCs upon the addition of 5 mm DA for 48 h. Insets are cartoons of the structure of two AgNCs (lower), and the photographs of their solution before (upper left) and after (upper right) the addition of 5 mm DA for 48 h. (K) Proposed formation mechanism of PDA/AgNC aerogels from AgNCs.

The assembly process was monitored by TEM imaging and in situ optical spectroscopy to further understand the assembly process of PDA/AgNC aerogels and elucidate the gelation mechanism of PDA/AgNC aerogels. As shown in Figure 3A, after adding DA for 1.5 h, AgNCs still dispersed well in the solution, but the particle size of AgNCs was 1.1 ± 0.5 nm (Figure S12), slightly larger than that of the prepared DHLA‐AgNCs (0.9 ± 0.3 nm). Upon extending the incubation time to 3 h, one can visualize their connection with each other to form short branched chain fragments in TEM images (Figure 3B). Meanwhile, the size of AgNCs in the composite increased to 1.4 ± 0.4 nm, suggesting the further growth of AgNCs during the assembly. As the assembly further evolved, relatively long chain fragments were obtained at 6 h (Figure 3C), and the network structure containing AgNCs gradually formed after 9 h (Figure 3D). When the reaction was further prolonged to 24 and 48 h, the network structure of the assembly became more compact and complete, and there was an obvious PDA layer on the surface of the final structures (Figure 3E,F). Quantitative analysis revealed that the thickness of the PDA layer is in the range of 1.0–1.3 nm. In addition, the ligament size of the gel network increased from 8.5 ± 2.0 nm to 10.3 ± 1.2 nm during 9–48 h (Figure 1E and Figure S13). Note that the size of individual AgNCs in the composite remained unchanged after 3 h (Figure 3G), indicating the subsequent process is mostly dominated by the gelation between PDA layers around nearby nanowire structures without the growth of AgNCs.

Due to the unique optical properties of AgNCs, the gelation process of AgNCs can be traced by UV–Vis absorption spectroscopy. As shown in Figure S14, the absorption spectrum of the reaction solution after adding DA for 1.5 h is similar to the typical absorption characteristics of AgNCs, and the characteristic absorption peak of DA at 280 nm appeared. Upon extending the incubation time from 3 to 9 h, the UV–Vis absorption spectra gradually evolved into a broad absorption band centered at around 500 nm owing to the presence of branched chain fragments formed by large‐sized AgNCs in the solution. When further extending the incubation time from 24 to 48 h, the scattering signal in the visible range significantly increased, indicating the formation of large assembly structures in the solution.^[^ ^27^ ^]^ Meanwhile, we noticed that the absorbance at 280 nm of the supernatant at 48 h was significantly lower than that at 24 h (Figure S15). This observation suggests the gradual consumption of DA from the solution during the gelation process, which is consistent with the increased thickness of the PDA layer observed in TEM.

PDA can be formed by oxidation‐induced polymerization of DA under alkaline conditions.^[^ ^35^ ^]^ In order to confirm whether the formation of the aerogel structure is the result of DA oxidation, the assembly of AgNCs by DA was carried out under argon protection (i.e., oxygen‐free). As shown in Figure S16, the color change of the solution was consistent with that in the air during the first 9 h. However, we didn't observe any obvious sedimentation after 24 h or longer time, which is different from that in the air. TEM image also showed that the obtained assembly under oxygen‐free conditions displays a preliminary network structure and there is no PDA layer observable on the surface (Figure S17). This result suggests that the network structure is mostly the result of interactions between DA and AgNCs, while further evolution of the gel structures is mainly driven by the adhesion of the surface PDA layers.

In order to further elucidate the role of DHLA as a ligand in the assembly process, the assembly of AgNCs protected by other ligands was investigated. Here, we chose AgNCs protected by short‐chain glutathione (GSH) and biomacromolecule lysozyme (Lys) as two representative ligands. UV–Vis absorption spectra in Figure 3H showed that GSH‐AgNCs and Lys‐AgNCs still retained the original characteristic absorption peaks of both AgNCs and DA. Indeed, TEM images also confirmed that both GSH‐AgNCs and Lys‐AgNCs did not form obvious network structures in the presence of DA (Figure 3I,J). Compared with DHLA, GSH and Lys have more abundant functional groups (e.g., amine) in their structures. Previous studies found that the strong binding force between DA and the substrates by the amine and catechol moieties interact synergistically to mediate surface priming by the catechol alkylamine compounds to material surfaces.^[^ ^36^ ^]^ The amines in DA may serve as molecular vanguards to allow the surface for bidentate catechol binding. Therefore, we speculate that amine groups in GSH and Lys may hinder the function of the amino groups in DA due to the repulsive forces. As a result, GSH‐AgNCs still exist in the system as individual particles, while Lys‐AgNCs will non‐specifically adsorb on the surface of PDA via weak interactions. Thus, the presence of rich carboxyl groups on the surface of AgNCs is believed to be another key factor for DA‐induced AgNCs assembly to form gel network structure.

Based on the above time‐lapse results and the characterization of PDA/AgNC aerogels, a possible assembly mechanism is proposed (Figure 3K): (1) AgNCs grow to larger particles and cluster together to form short branched chain fragments induced by DA; (2) the branched chain fragments are linked to form a preliminary network structure; (3) DA is oxidized and polymerized on the surface of the network structure to form a PDA layer; (4) the adhesion between the PDA layer on the surface of AgNC chains and the free PDA in the solution drives further cross‐link of the network, which gradually forms a 3D network structure.

### 2.3 Catalytic reduction property of PDA/AgNC aerogels

**FIGURE 4:**
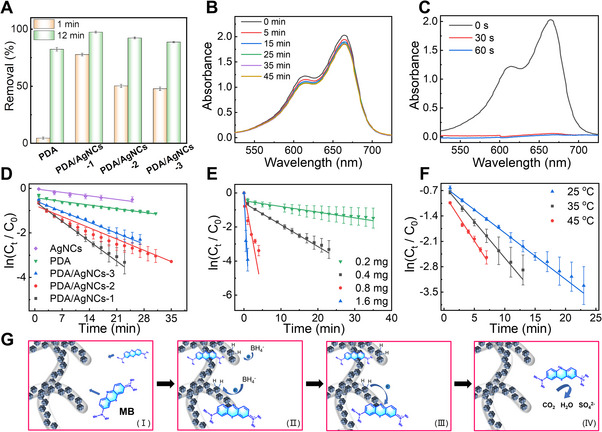
Characterization of dye degradation by PDA/AgNC aerogels. (A) Adsorption effect of different adsorbents to MB at 1 and 12 min. UV‐Vis absorption spectra of MB with NaBH_4_ in the (B) absence and (C) presence of 1.6 mg PDA/AgNCs‐1. (D) First‐order kinetics plot of the catalytic reduction of MB in the presence of NaBH_4_ and different catalysts. (E) First‐order kinetics plot of the catalytic reduction of MB in the presence of NaBH_4_ and different doses of PDA/AgNCs‐1. (F) First‐order kinetics plot of the catalytic reduction of MB in the presence of NaBH_4_ and PDA/AgNCs‐1 at different temperatures. (G) The proposed mechanism for MB reduction in the presence of NaBH_4_ by PDA/AgNCs. [Correction added on 9th July 2024, after first online publication: Figure 4 image was replaced with correct one.]

Considering the distinct porous structures of PDA/AgNC aerogels and the active catalytic activity of AgNCs,^[^ ^37^ ^]^ the potential application of the fabricated PDA/AgNC aerogels for catalytic reduction of methylene blue (MB), a model organic dye, was investigated. First, we studied the adsorption capability of PDA/AgNC aerogels towards MB, an essential step for the catalysts to exert efficient activity. Figure 4A displays the adsorption efficiency of as‐prepared PDA/AgNCs‐1, PDA/AgNCs‐2, PDA/AgNCs‐3 for MB at different times. As a comparison, the dye adsorption behavior of PDA particles was also investigated. Within 1 min incubation, all three PDA/AgNC aerogels exhibited a high adsorption efficiency above 47%, which is much larger than that of PDA (only 4.2%). Thanks to the three‐dimensional porous gel structure, PDA/AgNCs can provide large active surface and rich mass transfer pathways, so that MB can easily reach their surface. After 12 min, the adsorption rate of PDA particles increased to 82.2%, but the extent was still smaller than that of PDA/AgNC composites. Among the three aerogels, PDA/AgNCs‐1 possesses the highest adsorption efficiency, which may be due to the fact that it has the shortest ligament size and well‐defined pore structures. Recent studies revealed that Ag can also show a certain adsorption capacity of MB due to the electronic interactions,^[^ ^38^ ^]^ thus the relatively high Ag content of PDA/AgNCs‐1 may also contribute to its good adsorption performance.

PDA/AgNCs‐1 with the best dye adsorption performance was then selected to study the influence of the adsorbent amount on the adsorption performance. As shown in Figure S18, within the experimental range, the saturated adsorption amount of PDA/AgNCs‐1 slowly increased with raising the dosage until finally reaching equilibrium, approaching 50 mg g^−1^ when PDA/AgNCs‐1 is 0.4 mg. The adsorption isotherm experiment of PDA/AgNCs‐1 was also carried out to further understand its adsorption capacity. The adsorption data were fitted by both the Langmuir model and the Freundlich model (Figure S19). The fitting results showed that the adsorption process of PDA/AgNCs‐1 is more consistent with the Langmuir model, and the calculated maximum adsorption capacity was 50 mg g^−1^, which is consistent with the experimental results. The effect of pH on the adsorption properties of PDA/AgNC aerogels was also studied. As shown in Figure S20, with the increase of pH from 4.0 to 10.0, the adsorption rate of PDA/AgNCs for MB increased from 10.7% to 99.7%. This is mainly due to the increase of the electronegativity of the surface PDA layers at a higher pH, which promotes the electrostatic interactions between PDA and MB.^[^ ^38^ ^]^

As shown in Figure 4B, in the absence of the catalyst, the catalytic reduction effect of NaBH_4_ on MB is almost negligible. However, after adding PDA/AgNCs‐1, the absorbance at 664 nm decreased rapidly with time, and the reduction of MB was completed within 60 s (Figure 4C). The rate constant *k* of the catalytic reduction of MB by PDA/AgNCs‐1, PDA/AgNCs‐2, PDA/AgNCs‐3, PDA, and AgNCs was calculated as 0.13, 0.07, 0.06, 0.02, and 0.02 min^−1^, respectively (Figure 4D and Table S2). Compared with PDA and AgNCs, PDA/AgNC aerogels can not only capture MB to approach the material surface quickly but also provide abundant catalytic active sites within the 3D porous structures. Consequently, the reaction rate constants of PDA/AgNC composites are much larger than that of AgNCs and PDA alone. It is noteworthy that among three aerogels, PDA/AgNCs‐1 showed the best catalytic performance presumably due to its relatively small ligament size and high Ag/N ratio. Similar to the adsorption behavior of MB, the catalytic performance of PDA/AgNCs‐1 also exhibited a strong pH‐dependent behavior, as shown in Figure S21.

Next, the effect of the catalyst amount on the catalytic performance of PDA/AgNCs‐1 was investigated. As shown in Figure S22, the more catalyst dosage, the faster the reduction rate of MB. Through fitting the kinetic process, the value of *k* increased with increasing the amount of PDA/AgNCs‐1, and the maximum value of k reached 4.30 min^−1^ (Figure 4E and Table S3). As summarized in Table S4, the catalytic performance of PDA/AgNCs‐1, evaluated by the rate constant *k*_nor_ per unit catalyst mass, is better than that of most reported Ag nanocomposites.^[^ ^39^ ^]^ Moreover, as shown in Figure 4F and Table S5, the reaction rate significantly increased upon raising the reaction temperature, with the *k* increasing from 0.13 min^−1^ (25°C) to 0.26 min^−1^ (45°C). The activation energy (*E*_a_) calculated by the Arrhenius equation is 27.3 kJ mol^−1^ (Figure S23), which indicates that PDA/AgNCs‐1 has a significant effect on reducing the activation energy of the catalytic reduction reaction of MB.^[^ ^40^ ^]^ To further confirm the successful degradation of MB in the presence of PDA/AgNCs, the total organic carbon (TOC) analyzer was used to evaluate the organic matter content in the solution after the catalytic reaction. The TOC value was determined using the total concentration of organic matter in the solution, and the reduction in TOC reflected the degradation level near the end of the catalytic process. As shown in Figure S24, the decrease in TOC value further confirmed the successful degradation of MB.

For PDA/AgNC composites, their three‐dimensional network structure can provide a large specific surface area and rich materials transport pathways, which is conducive to the diffusion of both reducing agents and dyes toward the reactive surfaces. In addition, the adhesion property of the PDA layer on the surface of PDA/AgNCs also facilitates the adsorption of dye and reducing agents. Based on the reported catalytic mechanism of Ag nanomaterials,^[^ ^41^ ^]^ it is speculated that the catalytic mechanism of PDA/AgNCs composite aerogel is as follows (Figure 4G): (1) MB molecules are adsorbed on the surface of PDA/AgNCs and approach AgNCs due to electrostatic interaction and π‐π interaction with PDA; (2) electrons are transferred from NaBH_4_ to the surface of AgNCs and the Ag‐H intermediate is formed; (3) AgNCs transfer electrons to MB, then the electrons obtained by MB are reduced and separated from the surface. Particularly, the process of transferring electrons from donor species to dye receptors by Ag nanomaterials can lead to the structural destruction of MB molecules, which finally yields small molecules such as SO_4_^2−^, CO_2_, and H_2_O.^[^ ^41a^ ^]^

In order to investigate the applicability of PDA/AgNC aerogels in catalytic reduction of other common dyes, a heterocyclic cationic dye (e.g., Rhodamine 6G) and an azo anionic dye (e.g., Congo red) were selected. As seen in Figure S25, in the presence of PDA/AgNC aerogels, both dyes achieved nearly 100% reduction after 6 and 8 min, respectively, similar to that of MB. This result demonstrates the great potential of PDA/AgNC aerogels as an efficient catalyst for the treatment of organic dyes.

### 2.4 Antibacterial activity of PDA/AgNC aerogels

**FIGURE 5:**
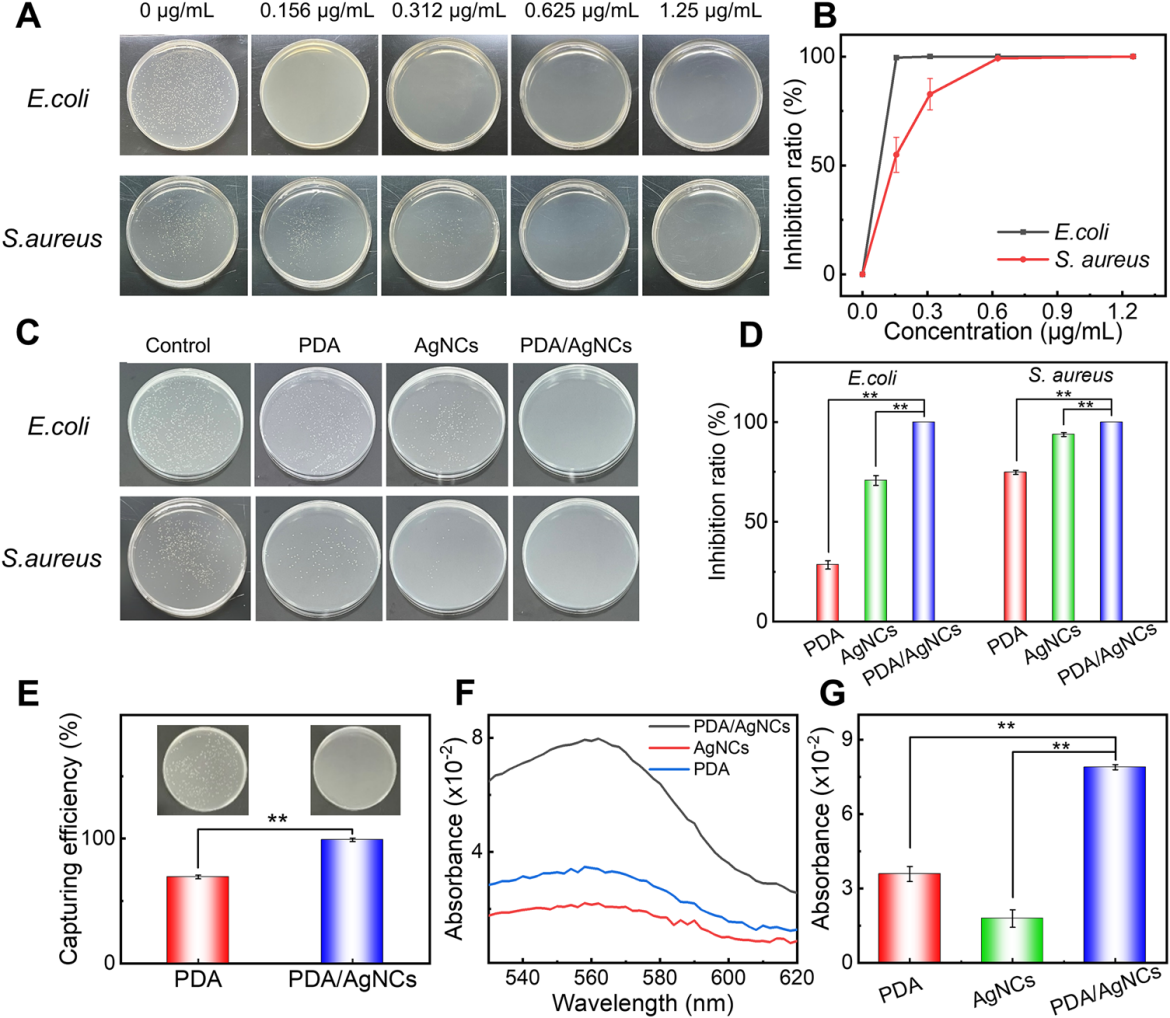
Antibacterial property and mechanism of PDA/AgNC aerogels. (A) Photographs of the antibacterial property of PDA/AgNCs‐1 at different concentrations on *E. coli* and *S. aureus* by the spread plate method. (B) The bacterial inhibition ratio of *E. coli* and *S. aureus* after being incubated with PDA/AgNCs‐1. (C) The antibacterial performance of PDA, AgNCs and PDA/AgNCs‐1 on *E. coli* and *S. aureus*. The concentration of PDA/AgNCs‐1 used for *E. coli* and *S. aureus* is 0.312 µg/mL and 1.25 µg/mL, respectively. (D) The bacterial inhibition ratio of *E. coli* and *S. aureus* after being incubated with PDA, AgNCs, or PDA/AgNCs‐1 determined by the spread plate method. (E) The capturing efficiency of *E. coli* by PDA and PDA/AgNCs‐1 (50 µg mL^−1^), and the corresponding photographs (inset). (F) UV–vis absorption spectra of *E. coli* treated with different materials (31 µg mL^−1^) based on BCA assay. (G) The statistics of the absorbance at 562 nm in (F). Asterisks represent statistically significant differences (*p < 0.05, **p < 0.01, ***p < 0.001).

Besides good catalytic properties, Ag nanomaterials are also known to exhibit strong bactericidal activity.^[^ ^42^ ^]^ Therefore, the antibacterial property of as‐prepared PDA/AgNC aerogels was investigated, with gram‐negative bacteria (*Escherichia coli*) and gram‐positive bacteria (*Staphylococcus aureus*) as the model strain. As seen in Figure 5A,B, the spread plate method showed that as low as 0.156 µg/mL PDA/AgNCs‐1 could kill over 99.5% of *E. coli*, while 0.625 µg/mL PDA/AgNCs‐1 could kill 99.2% of *S. aureus*. In stark contrast, a substantial number of bacterial colonies could still be observed for both PDA and AgNCs groups (Figure 5C). Quantitative analysis revealed that the sterilization rates of PDA/AgNCs against both bacteria are significantly higher than PDA and AgNCs (Figure 5D), suggesting remarkable, broad‐spectrum antimicrobial activity of PDA/AgNC aerogels. Herein, we note that the antibacterial performance of PDA/AgNCs also exceeds that of many reported silver nanocomposites, such as AgNPs@peptide/silk fibroin^[^ ^43^ ^]^ and Ag‐loaded zeolitic imidazolate framework‐8.^[^ ^44^ ^]^

The above results also implied that the presence of AgNCs contributed to the strong antibacterial properties of PDA/AgNC aerogels. Indeed, previous studies showed that AgNCs have broad‐spectrum antibacterial activities, and their antibacterial mechanism mainly involves damage to cell membranes, release of Ag^+^, production of reactive oxygen species (ROS), and destruction of intracellular components.^[^ ^45^ ^]^ Usually, the antibacterial mechanism of AgNCs is not singular, but rather a combination of multiple pathways. For example, the release of Ag^+^ is usually triggered by the oxidation of Ag^0^, which in turn can produce ROS and ultimately kill bacteria.^[^ ^46^ ^]^ Elevated levels of ROS can disrupt intracellular components such as proteins, enzymes, and DNA, thereby disrupting the normal metabolism and function of bacteria.^[^ ^47^ ^]^ Therefore, AgNCs in PDA/AgNCs are expected to play an important role in their antibacterial processes. Interestingly, compared to AgNCs, PDA/AgNC aerogels showed stronger antibacterial activity, as shown in Figure 5D. In order to understand the distinct antibacterial properties of PDA/AgNC aerogels, we selected *E. coli* as the representative bacteria to further explore their antibacterial mechanism. SEM images showed that PDA/AgNCs mostly adsorbed on the bacterial surface, and the treated *E. coli* appeared wrinkles and depressions (Figure S26), which is remarkably different from the control group. PDA is known to possess strong adhesion capability, which can efficiently adsorb to the surface of bacteria and destroy its structure, leading to bacterial death. According to previous reports, there are two main antibacterial mechanisms of PDA.^[^ ^48^ ^]^ One is that the catechol group of PDA can chelate the protein on the bacterial membrane of bacteria, affecting the normal physiological metabolism of bacteria leads to bacterial death. The other is caused by the abundant amines in PDA, where the positively charged amine groups will damage the cell wall and lead to the exudation of intracellular substances. Similarly, the presence of the PDA layer on the surface of PDA/AgNC aerogels will facilitate their contact with bacteria, further damaging the bacterial structure and leading to bacterial death.

Meanwhile, we note that the existence of a 3D porous network structure in PDA/AgNC aerogels also makes it easier to capture bacteria. In order to prove this conjecture, the capture ability of PDA and PDA/AgNCs‐1 to *E. coli* was then studied. As shown in Figure 5E, the capture efficiency of PDA/AgNCs‐1 to *E. coli* is 1.4 times that of PDA, indicating that PDA/AgNCs can capture bacteria and interact with bacterial surfaces more effectively than PDA. Moreover, the bicinchoninic acid assay (BCA) assay was used to investigate the leakage of bacterial protein. As shown in Figure 5F,G, PDA/AgNCs are indeed far more effective in destroying bacterial membranes than PDA and AgNCs.

### 2.5 Application of PDA/AgNC aerogels for water treatment

**FIGURE 6:**
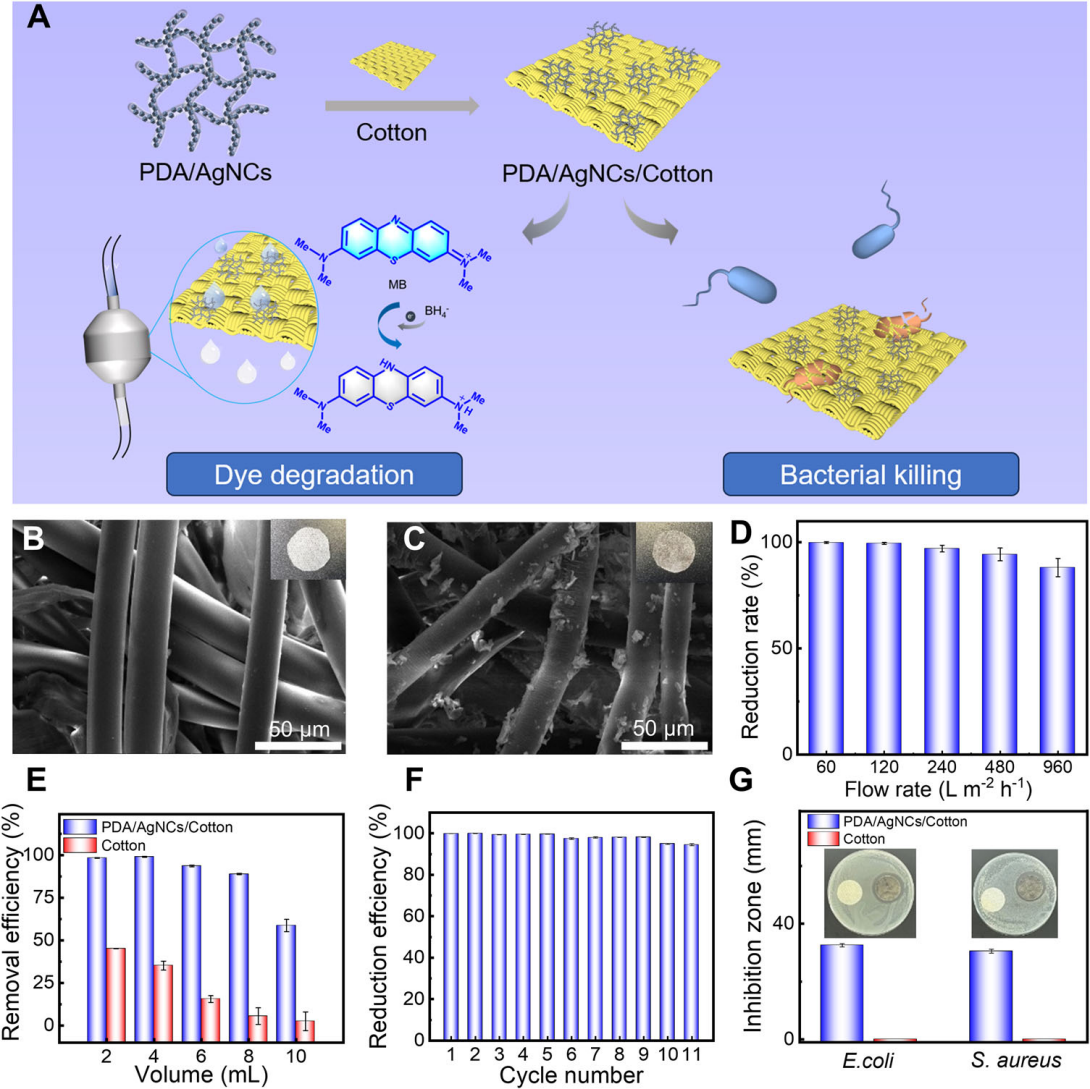
Application of PDA/AgNC aerogels for water treatment. (A) Schematic illustration of PDA/AgNCs‐functionalized cotton for water treatment. SEM images of (B) cotton and (C) PDA/AgNCs/cotton. Insets are the corresponding photographs. (D) The reduction efficiency of MB with PDA/AgNCs/cotton at different flow rates. (E) The reduction efficiency of MB with PDA/AgNCs/cotton and pristine cotton versus the filtration volume under a constant flux of 60 L m^−2^ h^−1^ in a flow‐through system. (F) The reduction efficiency of MB with PDA/AgNCs/Cotton versus the cycle number. (G) Inhibition zone size of PDA/AgNCs/cotton and pristine cotton on *E. coli* and *S. aureus*. Insets are the corresponding photographs: pristine cotton (left); PDA/AgNCs/cotton (right). [Correction added on 9th July 2024, after first online publication: Figure 6 image was replaced with correct one.]

The problem of water pollution caused by the discharge of dye wastewater has long been a serious threat to the environment. In addition, a large number of bacteria contained in the sewage will also harm human health and the environment.^[^ ^49^ ^]^ Although impressive progress has been made, it remains challenging to achieve efficient treatment of dyes and bacteria in wastewater simultaneously with the reported materials.^[^ ^50^ ^]^ Here, encouraged by the above results of PDA/AgNC aerogels, their potential utility for dye degradation and bacterial killing in water was then investigated. As‐prepared PDA/AgNC composites were loaded onto cotton by the impregnation method, named as PDA/AgNCs/Cotton (Figure 6A). As shown in Figure 6B,C, after PDA/AgNCs functionalization, the original smooth fiber of cotton became rather rough, confirming the successful loading of PDA/AgNC composites.

The flow catalytic performance of PDA/AgNCs/Cotton in the continuous filtration system was then carried out. As shown in Figure 6D, with the flow rate increasing from 60 L m^−2^ h^−1^ to 960 L m^−2^ h^−1^, the reduction efficiency of MB decreased from 99.8% to 88.1%. Apparently, the increase of the flow rate shortened the interaction time between MB solution and PDA/AgNCs/Cotton, thus decreasing the reaction efficiency. However, the reduction efficiency of MB was maintained to be higher than 88%, indicating a good catalytic activity of PDA/AgNCs/Cotton. Subsequently, a continuous flow catalytic reaction was carried out at a flow rate of 60 L m^−2^ h^−1^ to evaluate the sustainable catalytic performance of PDA/AgNCs/Cotton. After 8 mL of elution, the reduction efficiency of MB remained above 88% (Figure 6E). In stark contrast, for the unmodified cotton, the residual rate of MB was 45.1% in the initial 2 mL filtrate due to the MB adsorption in cotton, which then rapidly decreased to 5.5 % when the elution volume was increased to 8 mL. These results suggest that PDA/AgNCs/Cotton possess efficient capability to catalytically degrade the dyes under flowing conditions.

The long‐term catalytic performance of PDA/AgNCs/Cotton in the flow system was also studied. As shown in Figure 6F, the reduction efficiency of MB kept stably above 94% within 11 cycles, indicating robust stability and good reusability of PDA/AgNCs/Cotton. In order to further evaluate the stability of PDA/AgNCs on the cotton, the surface morphology of PDA/AgNCs/Cotton after 11 cycles were observed. As shown in Figure S27, the amount of PDA/AgNCs on the surface of cotton after 11 cycles only decreased slightly, suggesting good stability of PDA/AgNCs/Cotton during the long‐term catalytic application in flowing systems.

Then, the antibacterial activity of PDA/AgNCs/Cotton against *E. coli* and *S. aureus* was determined by the inhibition zone method. As shown in Figure 6G, an obvious inhibition zone around PDA/AgNCs/Cotton could be observed, while there was no visible inhibition zone around the pristine cotton, suggesting a good antibacterial activity of PDA/AgNCs/Cotton against *E. coli* and *S. aureus*. Further quantitative analysis revealed that the diameter of the inhibition zone of PDA/AgNCs/Cotton against *E. coli* and *S. aureus* was 32.6 mm and 30.5 mm, respectively, indicating that PDA/AgNCs/Cotton remained high broad‐spectrum bactericidal property of PDA/AgNC aerogels. Therefore, the as‐prepared PDA/AgNCs/Cotton not only possesses excellent catalytic activity in continuous flow systems but also exhibits good antibacterial activity, making them promising for water treatment in practical applications.

## 3 CONCLUSIONS

In summary, a new, mussel‐inspired approach for preparing 3D AgNC‐nucleated aerogels was developed based on DA‐induced self‐assembly. The obtained PDA/AgNC aerogels possess network structures comprised of nanowires with a uniform ligament size of 10.3 ± 1.2 nm, which is the smallest size among the reported Ag aerogels. Based on the characterization of the prepared aerogels and the monitoring of the gelation process, a unique gelation mechanism of AgNCs is proposed: AgNCs cluster together induced by DA to form a preliminary network structure, followed by oxidative polymerization of DA on the surface and further evolving into aerogels. Because of their highly porous structure, abundant AgNCs, and the synergistic effect of PDA, these aerogels exhibit robust stability and much enhanced catalytic/antibacterial activity compared with AgNCs. Furthermore, the proof‐of‐concept water‐treatment device based on PDA/AgNCs showed good performances in both catalytic dye degradation and bacterial killing, which may find promising applications in the environmental and healthcare fields. Particularly, the mussel‐inspired self‐assembly strategy proposed in this work has great potential in developing robust metal NC‐based functional materials, which also provides a new solution for designing sophisticated materials with integrated functions and synergistic properties.

## 4 EXPERIMENTAL SECTION

### 4.1 Materials

Lipoic acid (LA), silver nitrate (AgNO_3_), dopamine hydrochloride (DA), sodium borohydride (NaBH_4_), rhodamine 6G (R6G), and L‐glutathione reduced (GSH) were purchased from Sigma‐Aldrich (Shanghai, China). Sodium hydroxide (NaOH), nitric acid (HNO_3_), Congo red (CR), sodium chloride (NaCl), methylene blue (MB), cotton, and anhydrous ethanol were purchased from Sinopharm Chemical Reagent Co., Ltd. (Shanghai, China). Acetic acid was obtained from Shanghai Aladdin Bio‐Chem Technology Co., Ltd. Lysozyme (Lys) was purchased from Alsace Biotechnology Co., Ltd (Xi'an, China). Bicinchoninic acid assay (BCA) protein concentration determination kit was purchased from Biyuntian Biotechnology Co., Ltd (Xi'an, China). Tryptone, agar, anhydrous sodium dihydrogen phosphate (NaH_2_PO_4_), and anhydrous disodium hydrogen phosphate (Na_2_HPO_4_) were obtained from Sangon Biotech (Shanghai, China). *S. aureus* and *E. coli* were obtained from BeNa Culture Collection. Yeast was purchased from Beijing Aoboxing Biotechnology Co., Ltd. All the chemicals and reagents were at least analytical grade and used as received without further treatment. All solutions were prepared with deionized water (18.25 MΩ cm) throughout the whole experiment.

### 4.2 Synthesis of AgNCs

Dihydrolipoic acid‐stabilized AgNCs (DHLA‐AgNCs) were synthesized by modifying a reported method.^[^ ^15^ ^]^ LA (304 mg) was solubilized in an aqueous solution (75 mL) containing NaOH (2 m, 900 µL), and AgNO_3_ (0.2 m, 500 µL) was added. After stirring for 5 min, freshly prepared NaBH_4_ (0.12 m, 4 mL) was added to the mixture under rapid stirring. The reaction was stopped after stirring for 15 h in the dark, and the solution was stored in the dark at 4°C for later use. As a comparison, GSH‐stabilized AgNCs (GSH‐AgNCs) and Lys‐stabilized AgNCs (Lys‐AgNCs) were also synthesized by the reported methods,^[^ ^51^ ^]^ and stored at 4°C in the dark.

### 4.3 Synthesis of PDA particles

PDA particles were prepared according to the previous report.^[^ ^52^ ^]^ DA (20 mg) was dissolved in NaOH (0.6 mm, 40 mL) solution. Then the black PDA suspension was obtained after magnetic stirring for 18 h. PDA particles were then separated by centrifugation, washed with deionized water several times, and finally freeze‐dried for further use.

### 4.4 Fabrication of PDA/AgNC aerogels

Before the assembly, the AgNC solution was first purified using ultracentrifuge filters with H_2_O as the solvent. To initiate the assembly of AgNCs, the pH of the DHLA‐AgNCs solution (990 µL) was adjusted to 10.0, followed by the addition of DA (10 µL) with different concentrations (Final concentration of DA is 0, 2.5, 5, 10, and 20 mm). Then, the solution was placed in the dark at 35°C for gelation. Similarly, DA‐triggered hydrogels of GSH‐AgNCs and Lys‐AgNCs were also prepared by the same procedure as above (5 mm DA, 35°C). The as‐prepared hydrogel was washed with water six times before solvent exchange with tert‐butanol. After being flash‐frozen and freeze‐dried for 24 h, the corresponding aerogel was obtained.

### 4.5 Preparation of PDA/AgNC‐loaded cotton

Regularly cut cotton (diameter: 25 mm) was soaked in the mixed solution of ethanol and acetone under ultrasonic cleaning for 30 min. Then the cotton was immersed in PDA/AgNCs suspension under shaking for 12 h. Afterward, PDA/AgNCs functionalized cotton was thoroughly rinsed with deionized water and dried in the air.

### 4.6 Adsorption performance of PDA/AgNC aerogels

The adsorption tests of MB on PDA/AgNCs aerogels were carried out as the following procedure. Adsorbents were added to MB solution (16 mg L^−1^) under shaking at different times. Then the supernatant was removed after the centrifugation. The concentration of MB in the supernatant was quantified by UV–vis absorption spectroscopy.

### 4.7 Catalytic performance of PDA/AgNC aerogels

The catalytic properties of the as‐synthesized PDA/AgNCs aerogels were investigated using the reduction of MB by NaBH_4_ as a model system. In a typical test, PDA/AgNCs (0.4 mg) were added to MB solution (16 mg L^−1^, 3.88 mL). Then, fresh NaBH_4_ solution (0.1 m, 0.12 mL) was injected into the solution under shaking. The catalytic process was monitored by measuring the changes in the absorbance at 664 nm.

### 4.8 Antibacterial experiments

The antibacterial activity of PDA/AgNCs against *S. aureus* and *E. coli* was quantitatively evaluated by the spread plate method. The bacteria were cultured in the sterile liquid medium (1 g tryptone, 0.5 g yeast, 1 g NaCl in 100 mL deionized water). Then, the bacterial suspension (10^6^ CFU mL^−1^, 200 µL) was incubated with PBS (control), PDA/AgNCs, AgNCs, or PDA at 37°C for 6 h. Then the bacterial suspension was diluted to 10^4^ CFU mL^−1^, and the diluted bacterial suspension (100 µL) was spread on a standard agar medium. Next, the surviving bacteria were incubated at 37°C for 12 h, and the bacterial colonies were counted.

To investigate the bacteria capturing ability, bacteria (1 × 10^6^ CFU mL^−1^, 100 µL) were mixed with PBS (control group), PDA/AgNCs, PDA, or AgNCs (100 µg mL^−1^, 100 µL) separately. After standing for 10 min, the supernatant was diluted to 10^4^ CFU mL^−1^, and the diluted supernatant (100 µL) was spread on a standard agar medium. Next, the surviving bacteria were incubated at 37°C for 12 h, and the bacterial colonies were counted.

The protein leaked by bacteria was tested by the BCA method. Bacteria (1 × 10^6^ CFU mL^−1^, 300 µL) was mixed with PBS (control group), PDA/AgNCs, PDA, or AgNCs (62 µg mL^−1^, 300 µL) separately. After incubating at 37°C for 6 h, the solution was centrifuged at 10,000 rpm for 8 min. The supernatant was removed and BCA working solution (200 µL) was added. After incubating at 37°C for 80 min, the solution was measured by the microplate reader.

### 4.9 Catalytic reduction of MB using PDA/AgNCs/cotton

The prepared PDA/AgNCs/Cotton was inserted into a 25 mm diameter polypropylene filter for flow catalysis experiments. The MB solution containing NaBH_4_ was used as the feed solution. The solution (2 mL) after flowing through the PDA/AgNCs/cotton was taken to measure the absorbance at 664 nm. In the following test cycles, wash the cotton with water and ethanol, and then add fresh feed solution to evaluate the catalytic activity of the material in the same way.

### 4.10 Antibacterial test of PDA/AgNCs/cotton

An agar diffusion test was used to detect the inhibition zone. The diluted bacteria *(S. aureus* or *E. coli*, 100 µL, 1 × 10^6^ CFU mL^−1^) were uniformly spread onto an agar plate using a coating rod. Then the pristine cotton and PDA/AgNCs/cotton were placed in the middle of the agar plate, separately. After incubation at 37°C for 24 h, the diameter of the inhibition zone was measured.

## AUTHOR CONTRIBUTIONS

Yunshan Gao designed and conducted the experiments, and wrote initial drafts of the manuscript. Jie Xu assisted with the materials preparation and data analysis. Shaohua Qu assisted in the characterization of materials. Yixiao Li assisted in the antibacterial experiment. Gleb B. Sukhorukov assisted with the analysis and writing. Li Shang conceived the project, supervised the experiment, and finalized the manuscript.

## CONFLICT OF INTEREST STATEMENT

The authors declare no conflicts of interest.

## Supporting information

Supporting Information

## Data Availability

The data that support the findings of this study are available from the corresponding author upon reasonable request.
