# Supplementary material for: Mussel‐inspired self‐assembly of silver nanoclusters into multifunctional silver aerogels for enhanced catalytic and bactericidal applications
Source: Exploration (Beijing). 2024 Jun 26;5(1):20240034. doi: 10.1002/EXP.20240034 (PMC11875448; doi:10.1002/EXP.20240034)
Supplement: Supplementary file 1 — Supporting Information [file EXP2-5-20240034-s001.pdf]

Supporting Information

Mussel-inspired Self-assembly of Silver Nanoclusters into Multifunctional Silver Aerogels for Enhanced Catalytic and Bactericidal Applications

*Yunshan Gao, Jie Xu, Shaohua Qu, Yixiao Li, Gleb B. Sukhorukov, Li Shang**

Y. Gao, Dr. J. Xu, Dr. S. Qu, Dr. Y. Li, Prof. Dr. L. Shang
State Key Laboratory of Solidification Processing, School of Materials Science and Engineering, Northwestern Polytechnical University, Xi'an, 710072, China.
E-mail: li.shang@nwpu.edu.cn

Prof. Dr. G.B. Sukhorukov
Skolkovo Institute of Science and Technology, Bolshoi pr.30, Moscow 143025, Russia

Supplementary Experimental Section

Material characterization: UV-vis absorption spectra were collected using a UV-Vis spectrophotometer (U-3900H, Hitachi, Tokyo, Japan). Morphology of PDA/AgNCs was observed using a scanning electron microscope (SEM, FEI Nano SEM 450) and a Talos F200X transmission electron microscope (TEM, FEI, USA). TEM samples are obtained by dispersing them in water and then dropping them onto carbon-coated copper grids. Based on TEM images, the size distribution of AgNCs and the ligament size of PDA/AgNC aerogel were measured using Nano Measurer software. X-ray photoelectron spectroscopy (XPS) was performed on an Axis Ultra DLD XPS spectrometer (Kratos, Manchester, UK), using Al Kα X-ray radiation (1486.6 eV) for excitation. The Fourier transform infrared (FT-IR) spectra were measured with a Thermo Scientific Nicolet iS50 FT-IR spectrometer. Thermogravimetric analysis (TGA) was measured on a simultaneous thermal analyzer (STA 449F3) in the temperature range of 35-800 ℃ at a heating rate of 10 ℃ min^{-1}. AgNCs and PDA/AgNCs need to be freeze-dried in a freeze-drying device (SCIENTZ-10ND) for XPS and TGA measurements. Nitrogen physisorption isotherms were measured at 77 K on a Micromeritics ASAP 2460 instrument. The specific surface area was calculated by using the multi-point Brunauer-Emmett-Teller (BET) equation. The pore size was evaluated using the quenched solid density functional theory (QSDFT) equilibrium model and the total pore volume was calculated using the Barrett-Joyner-Halenda (BJH) method.

Adsorption performance of PDA/AgNCs: The concentration of MB in the solution was quantified by UV-vis absorption spectroscopy, and the calibration curve of absorbance vs. MB concentration was first established. The adsorption capacity of the as-prepared PDA/AgNCs-1 was calculated based on the adsorption isotherm experiment, which was carried out at room temperature for 24 h to reach adsorption equilibrium. The equilibrium adsorption amount (Q_e) was calculated by the Equation 1:

$$Q_e = \frac{(C_0 - C_e)\ V}{m} \quad (1),$$

where C_0 is the initial concentration of MB solution, C_e is the equilibrium concentration of MB solution after 24 h adsorption by PDA/AgNCs-1, V is the volume of MB solution, and m is the mass of PDA/AgNCs-1. The adsorption efficiency of MB by PDA/AgNC aerogels and PDA can be determined by the Equation 2:

$$R = \frac{C_0 - C_t}{C_0} \times 100\% \quad (2),$$

where C_0 is the initial concentration of MB, C_t is the concentration of MB solution at t time.

Langmuir model and Freundlich model are the most commonly used adsorption isotherm models. The Langmuir model and the Flemish model are shown as Equation 3 and 4, respectively:

$$\frac{C_e}{q_e} = \frac{1}{q_m b} + \frac{1}{q_m} C_e \quad (3);$$

$$Inq_e = InK_f + \frac{1}{n} InC_e \quad (4),$$

where C_e is the adsorption equilibrium concentration of dye (mg/L), q_e is the equilibrium adsorption amount (mg/g), q_m is the calculated saturated adsorption capacity (mg/g), b is Langmuir constant related to adsorption energy (L/mg), K_f and n are Freundlich constants related to adsorption energy.

Catalytic performance of PDA/AgNCs: The catalytic properties of the as-synthesized PDA/AgNC aerogels were investigated using the reduction of MB by $NaBH_4$ as a model. In order to characterize the catalytic effect of the catalyst, the catalytic process was dynamically fitted. Since the concentration of $NaBH_4$ is in a great excess, which can be considered as constant during the reaction, the reduction of MB can be assumed to follow pseudo-first-order kinetics (Equation 5). The catalytic reduction ratio was calculated by the Equation 6:

$$-In(\frac{C_t}{C_0}) = kt \quad (5);$$

$$R = \frac{C_0 - C_t}{C_0} \times 100\% \quad (6),$$

where k is the reaction rate constant, C_0 is the initial concentration of MB, and C_t is the concentration of MB at a reaction time of t.

The activation energy (E_a) is an empirical parameter reflecting the rate constant of the catalytic reaction related to the temperature. The Arrhenius equation can be described as:

$$Ink = InA + \frac{E_a}{RT} \quad (7)$$

where k is the rate constant at temperature T (Kelvin), A is the Arrhenius factor, and R is the general gas constant.

Antibacterial activity assay: The antibacterial ratio was calculated using the following formula:

$$\text{Survival rate (\%)} = \frac{\lambda_{sample}}{\lambda_{control}} \times 100\% \quad (8);$$

$$\text{Antibacterial efficiency (\%)} = \frac{\lambda_{control} - \lambda_{sample}}{\lambda_{control}} \times 100\% \quad (9).$$

The bacterial capture rate was calculated by the following equation:

$$\text{Capture rate (\%)} = \frac{\lambda_{control} - \lambda_{sample}}{\lambda_{control}} \times 100\% \quad (10),$$

where $\lambda_{control}$ is the number of bacterial colonies in the control group, and λ_{sample} is the number of bacterial colonies in the sample group.

Supplementary Figures

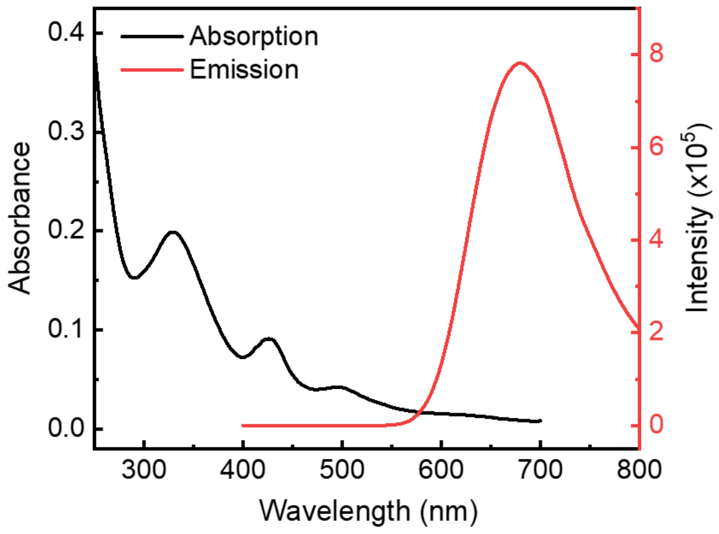

Figure S1. UV-Vis absorption (black) and fluorescence emission (red, Ex=340 nm) spectra of DHLA-AgNCs in aqueous solution.

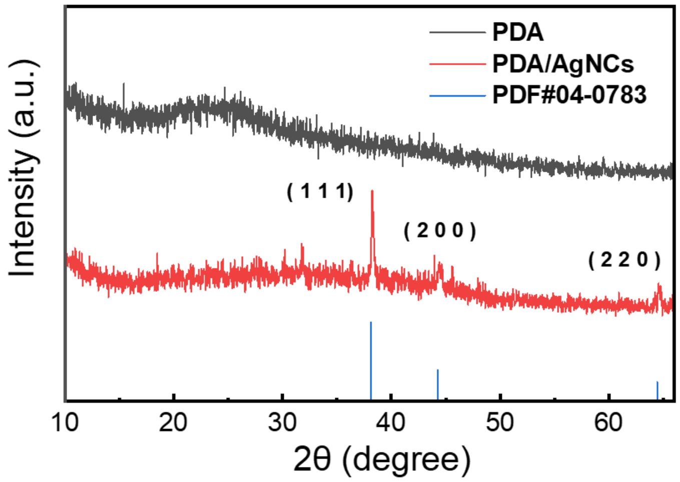

Figure S2. XRD patterns of PDA and PDA/AgNCs.

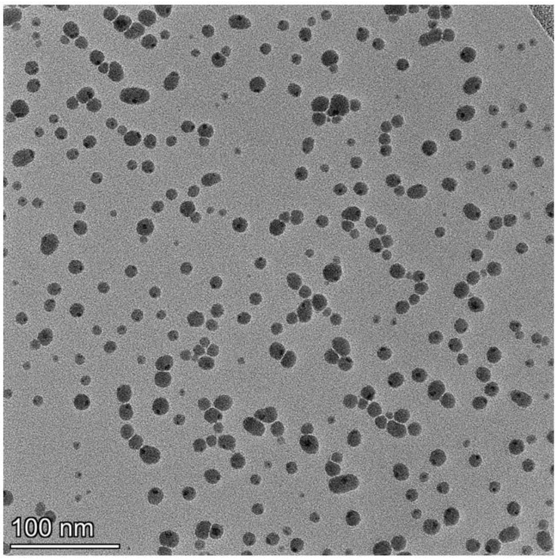

Figure S3. TEM image of purified DHLA-AgNCs solution in the absence of DA.

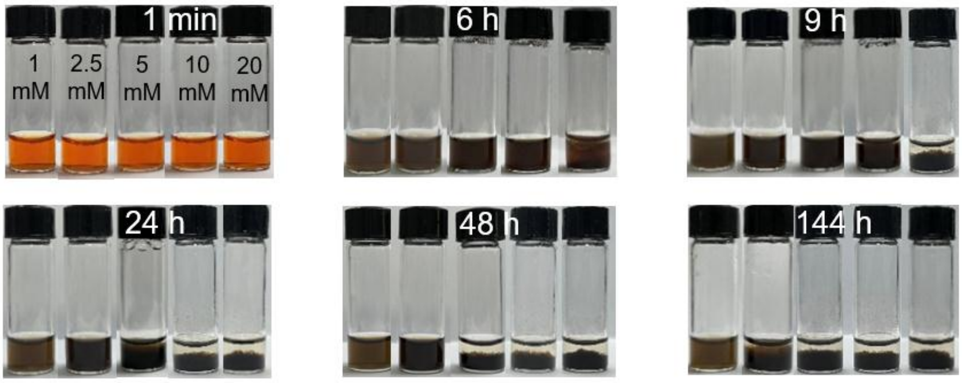

Figure S4. Time-lapse photos of PDA/AgNC solution obtained by different initial DA concentrations (from left to right): 1 mM, 2.5 mM, 5 mM, 10 mM and 20 mM.

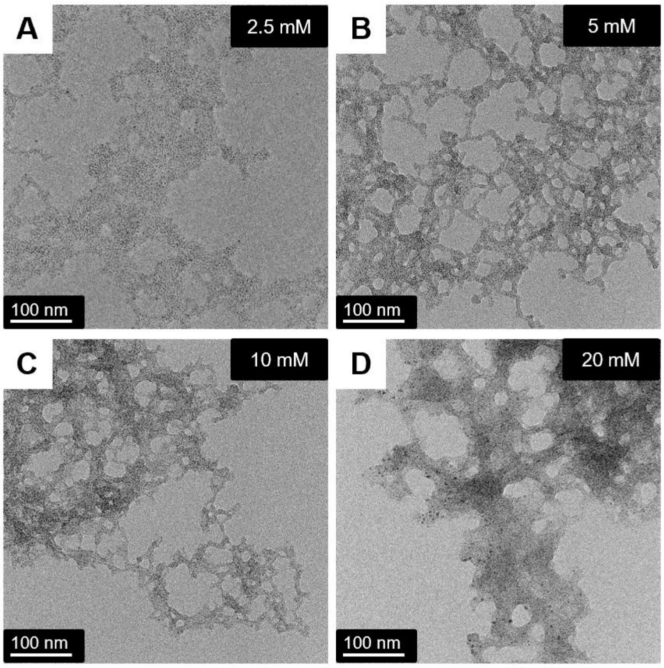

Figure S5. TEM images of PDA/AgNCs obtained by different initial DA concentrations: (A) 2.5 mM, (B) 5 mM, (C) 10 mM and (D) 20 mM.

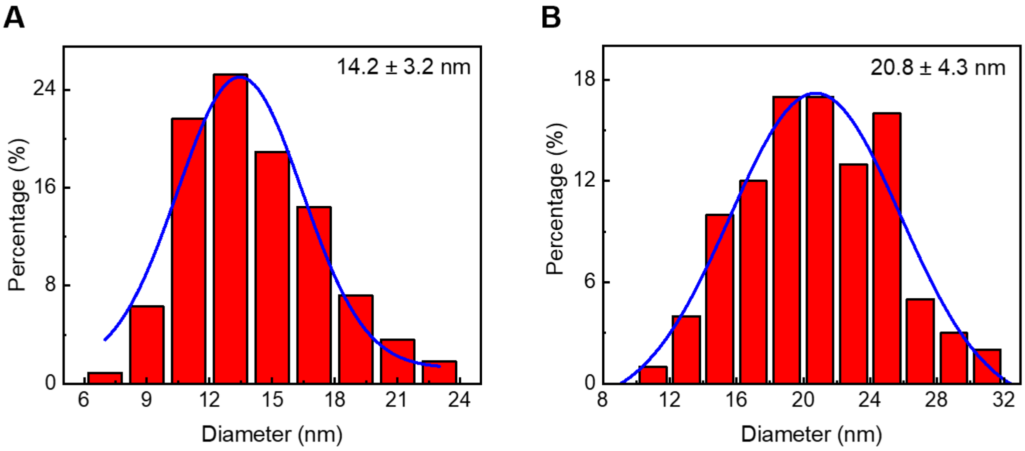

Figure S6. The ligament size distribution of PDA/AgNCs obtained by (A) 10 mM and (B) 20 mM DA, based on TEM images.

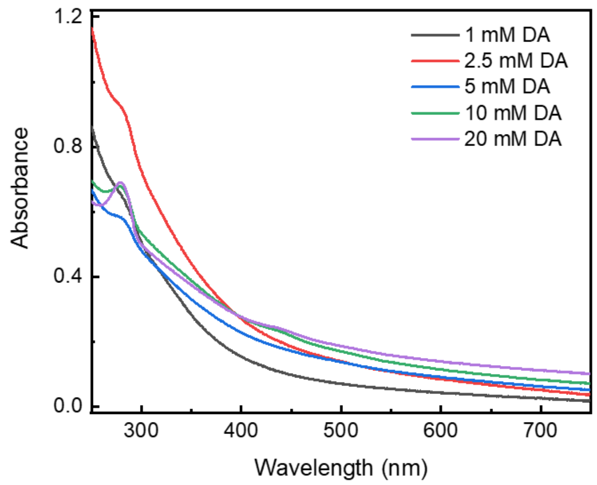

Figure S7. UV-Vis absorption spectra of PDA/AgNCs obtained by different DA concentrations.

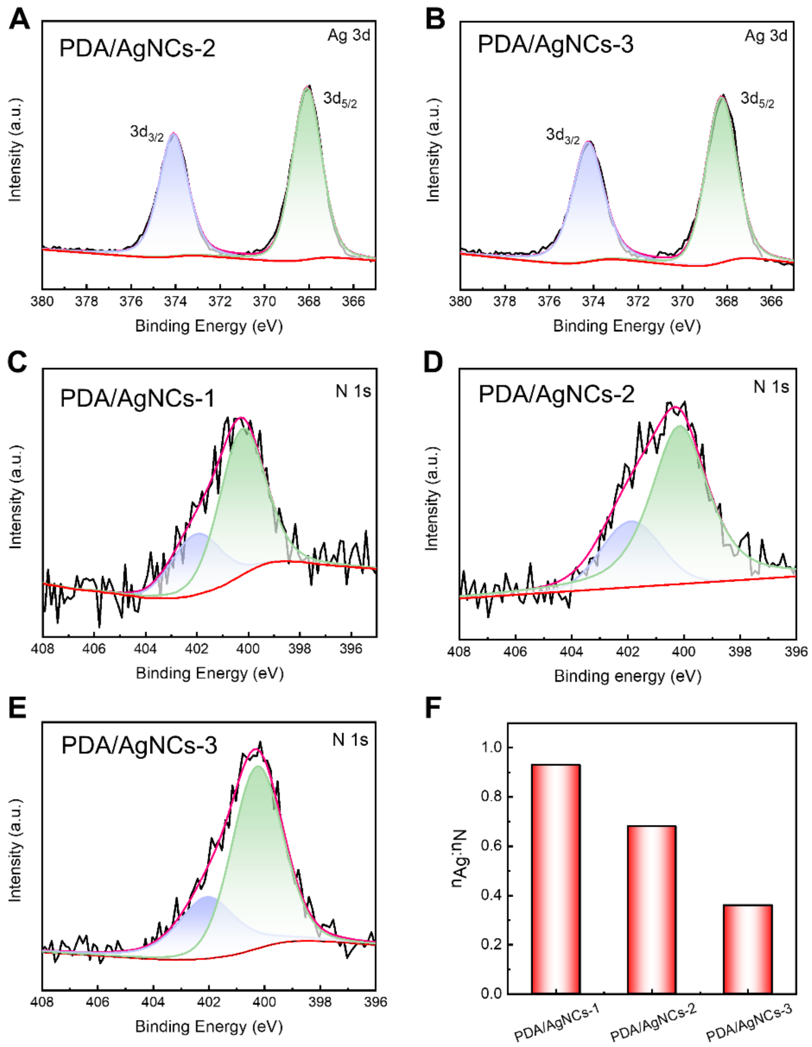

Figure S8. XPS spectra of the Ag 3d for (A) PDA/AgNCs-2 and (B) PDA/AgNCs-3. XPS spectra of N 1s for (C) PDA/AgNCs-1, (D) PDA/AgNCs-2 and (E) PDA/AgNCs-3. (F) The ratio of Ag to N for PDA/AgNCs-1, PDA/AgNCs-2 and PDA/AgNCs-3.

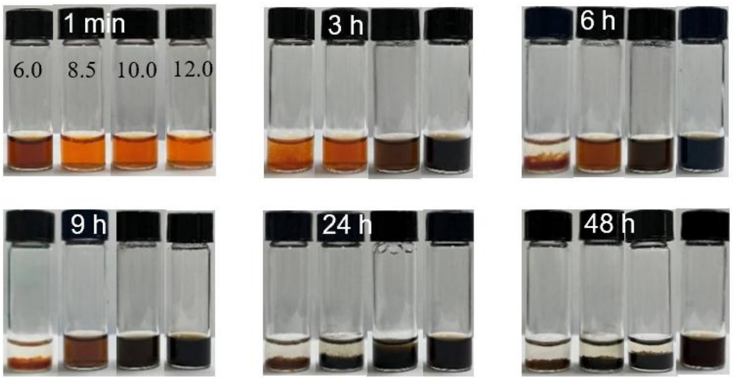

Figure S9. Time-lapse photos of the PDA/AgNC solution induced by 5 mM DA under different pH conditions.

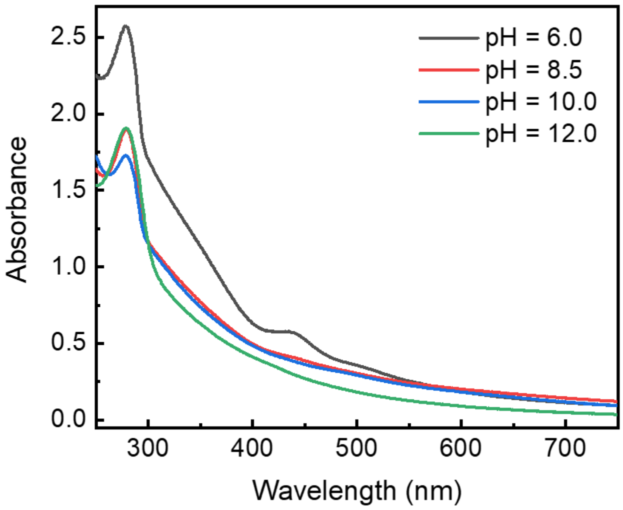

Figure S10. UV-Vis absorption spectra of PDA/AgNC solution obtained by 5 mM DA under different pH conditions.

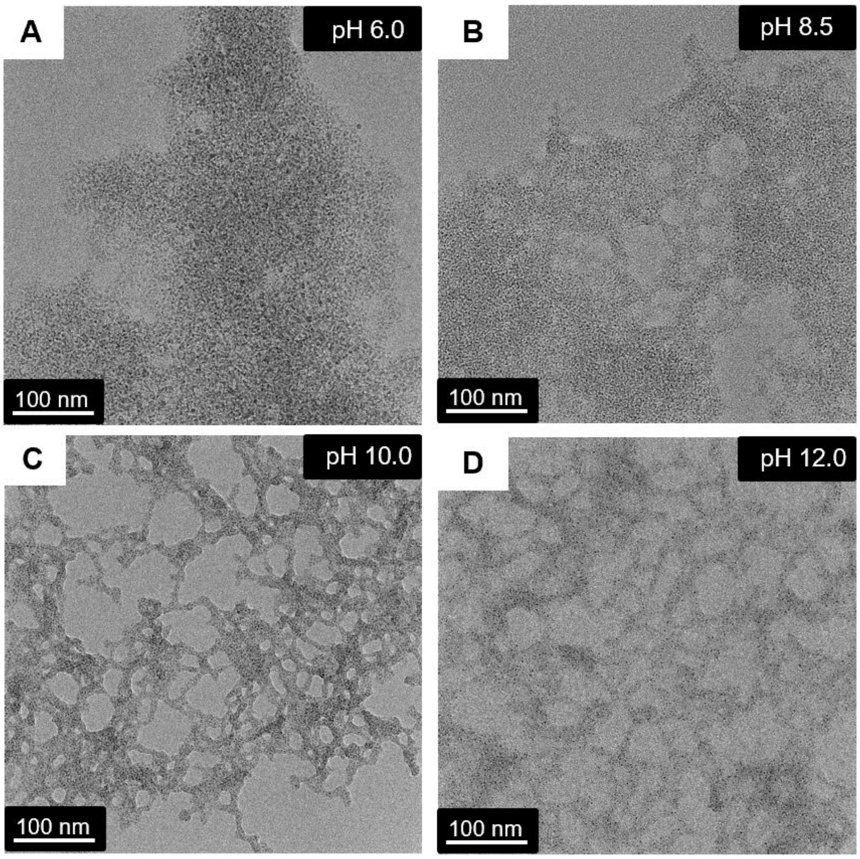

Figure S11. TEM images of PDA/AgNCs obtained by 5 mM DA under different pH: (A) 6.0, (B) 8.5, (C) 10.0 and (D) 12.0.

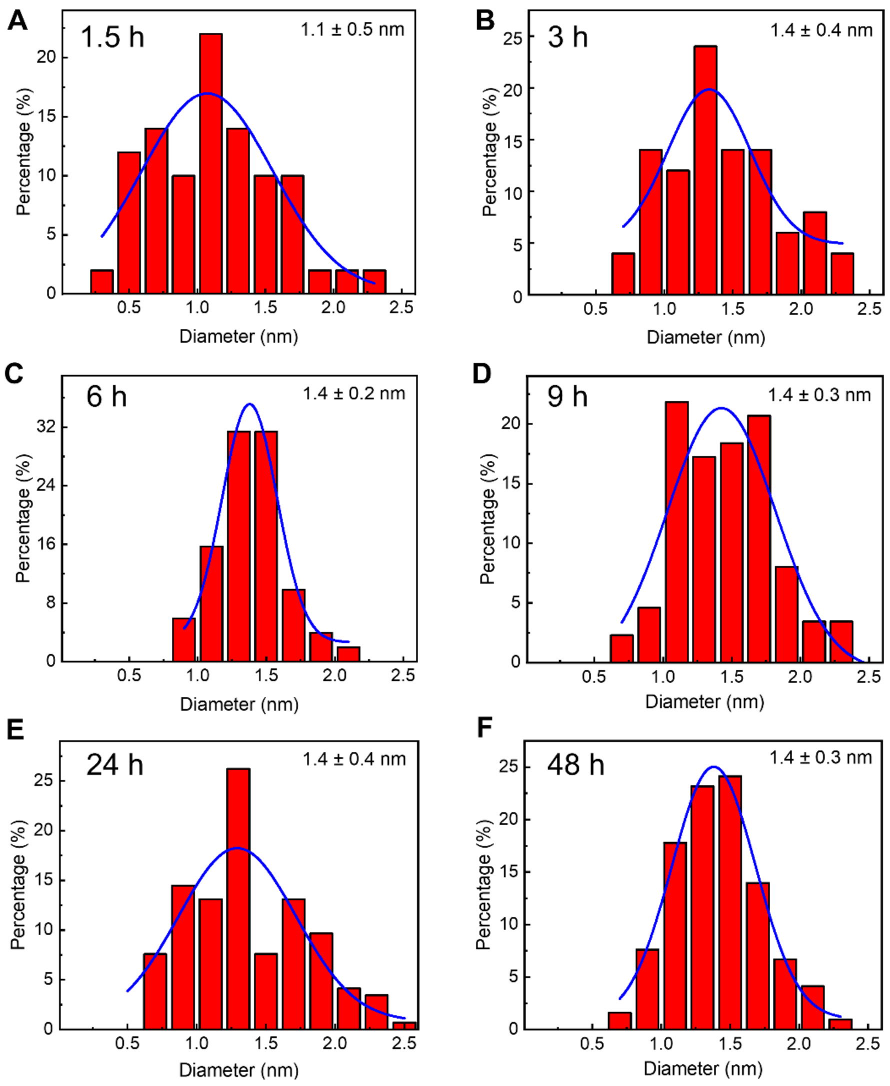

Figure S12. Size histograms of the AgNC assembly process at different timepoints: (A) 1.5 h, (B) 3 h, (C) 6 h, (D) 9 h, (E) 24 h and (F) 48 h, in the presence of 5 mM DA.

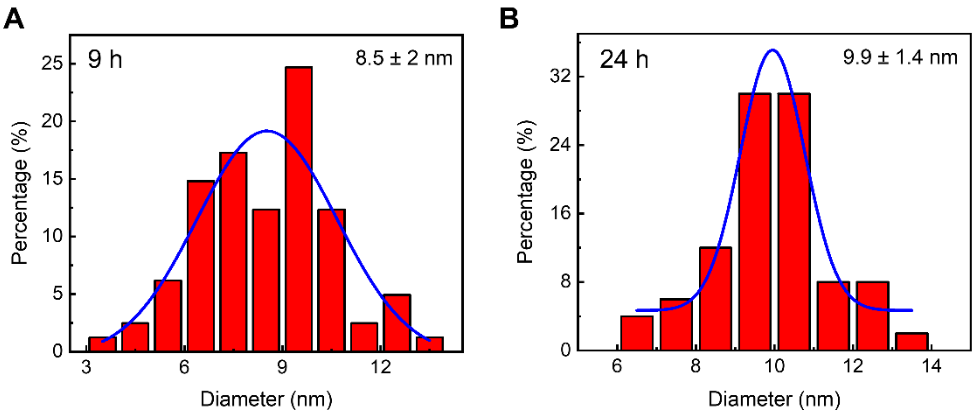

Figure S13. The corresponding ligament size distribution of the PDA/AgNC assembly obtained at (A) 9 h and (B) 24 h, in the presence of 5 mM DA.

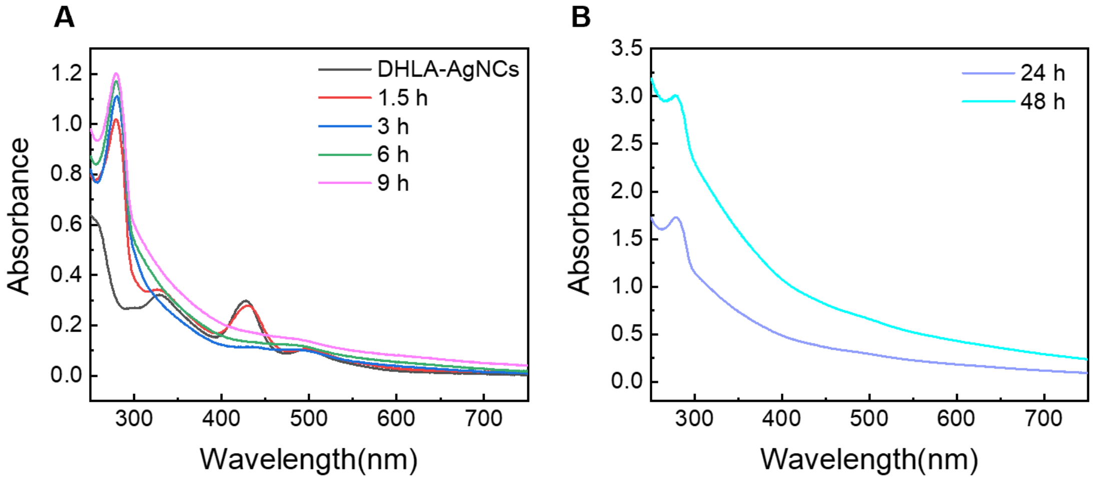

Figure S14. Time-lapse UV-Vis absorption spectra of the assembly process of PDA/AgNCs, upon adding 5 mM DA: (A) 0-9 h; (B) 24-48 h.

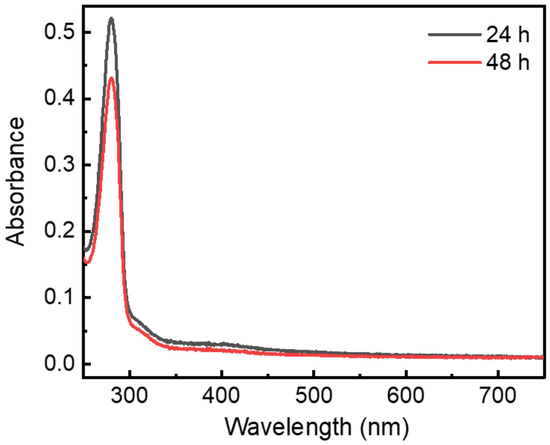

Figure S15. UV-Vis absorption spectra of the supernatant of the PDA/AgNC assembly obtained at 24 h and 48 h, in the presence of 5 mM DA.

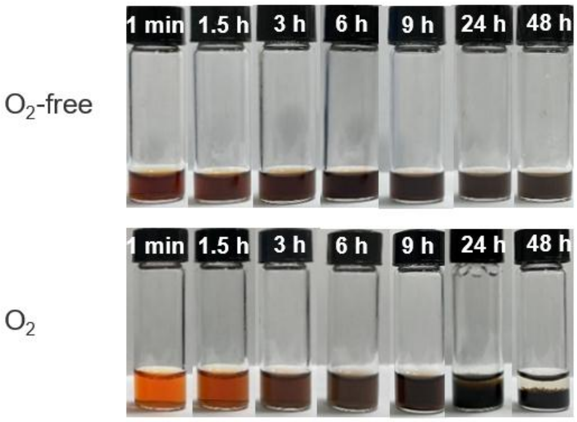

Figure S16. Time-lapse photos of the assembly process of PDA/AgNCs in the absence (upper) and presence (bottom) of oxygen, in the presence of 5 mM DA.

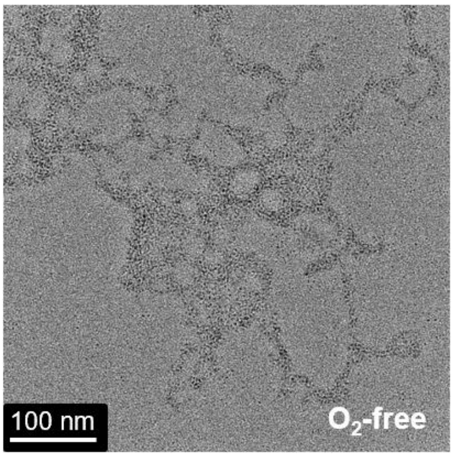

Figure S17. TEM image of the AgNCs assembly at 48 h upon the addition of 5 mM DA in the absence of oxygen.

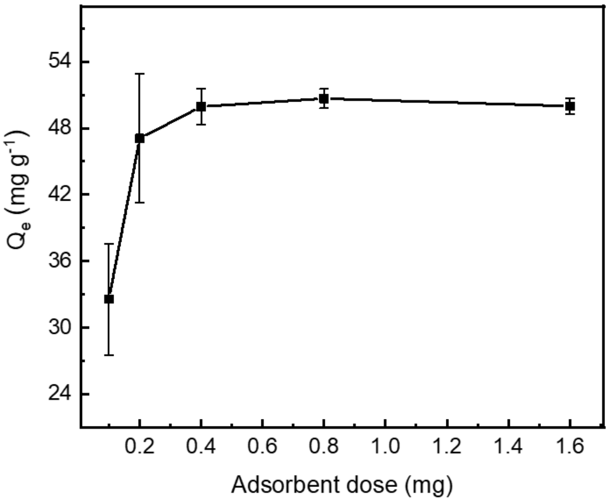

Figure S18. Effect of the dosage on the saturated adsorption capacity of PDA/AgNCs-1.

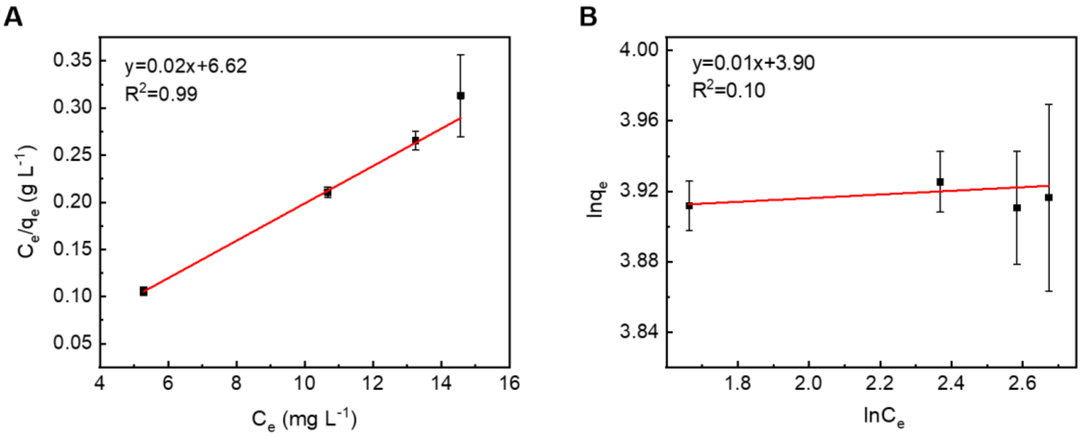

Figure S19. Linear fitting of the dye adsorption data by two isotherm models: (A) Langmuir; (B) Freundlich.

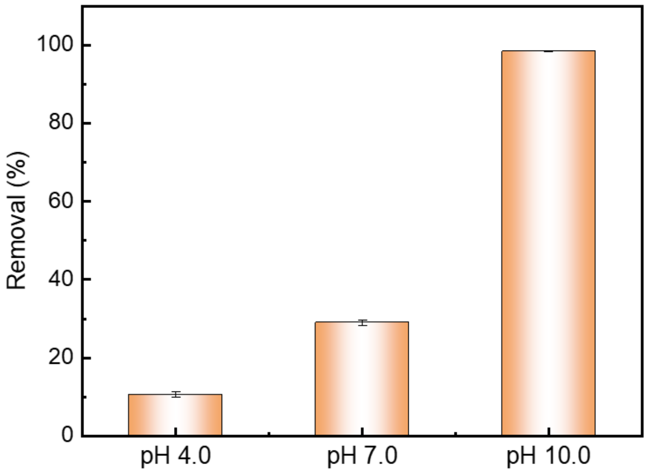

Figure S20. Effect of pH on the adsorption property of PDA/AgNCs-1.

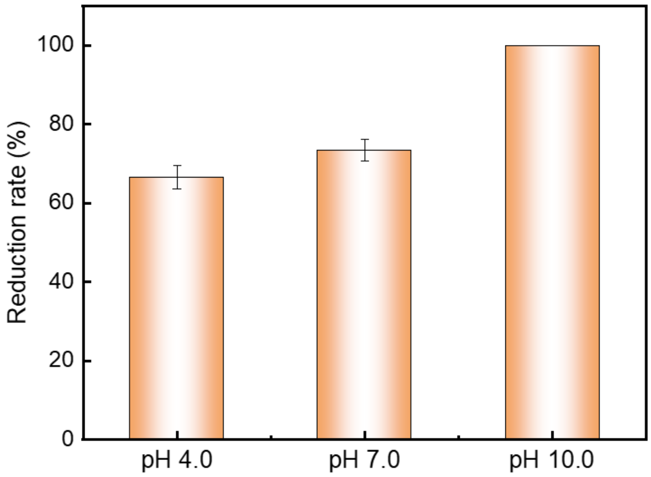

Figure S21. Catalytic performance of PDA/AgNCs-1 for the reduction of MB by $NaBH_4$ under different pH conditions.

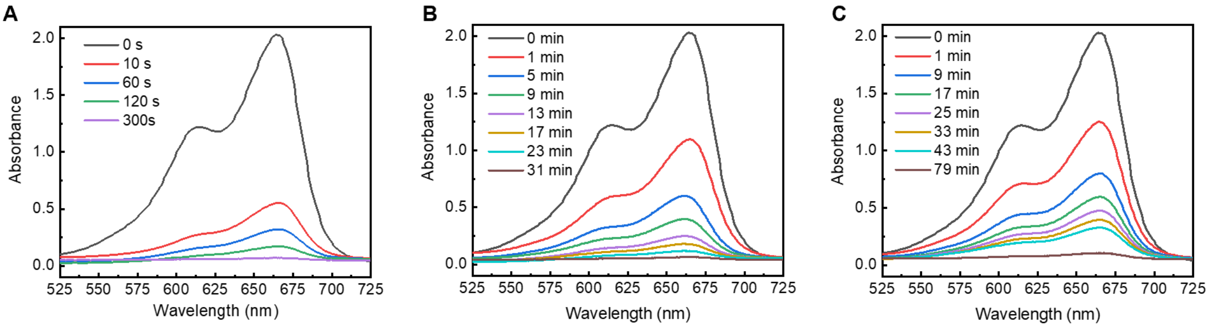

Figure S22. UV-Vis absorption spectra of MB with $NaBH_4$ in the presence of different doses of PDA/AgNCs-1: (A) 0.8 mg, (B) 0.4 mg, (C) 0.2 mg.

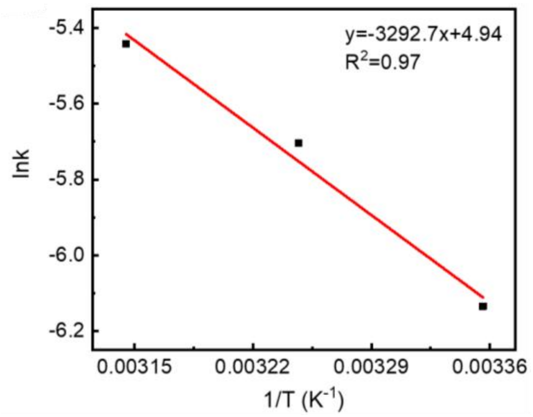

Figure S23. Arrhenius diagram of lnk versus 1/T of the catalytic reduction of MB in the presence of $NaBH_4$ and PDA/AgNCs-1 at different temperatures.

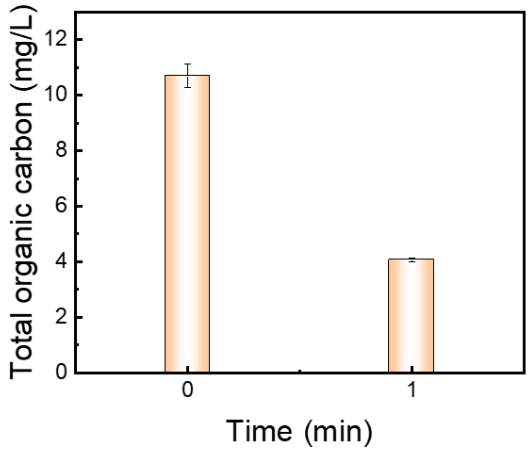

Figure S24. TOC value for MB with $NaBH_4$ in the presence of 1.6 mg PDA/AgNCs-1.

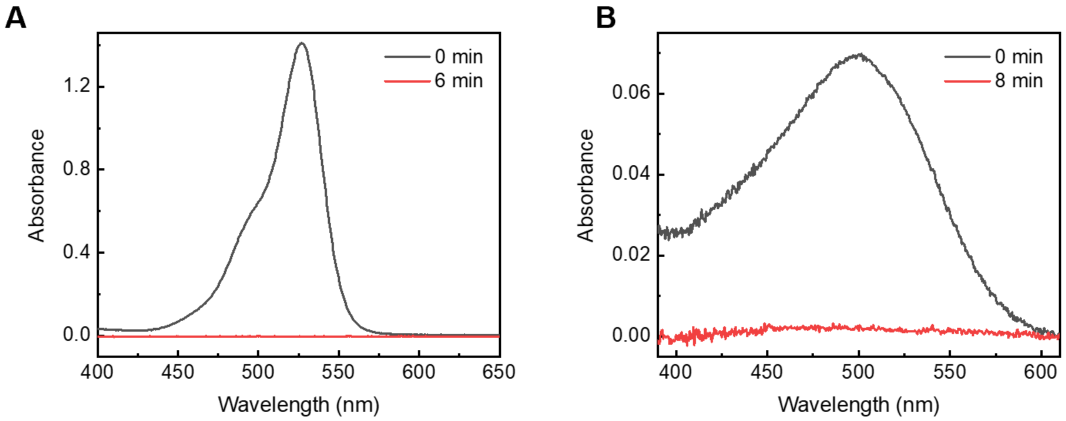

Figure S25. Catalytic reduction results of (A) R6G and (B) CR by PDA/AgNCs-1.

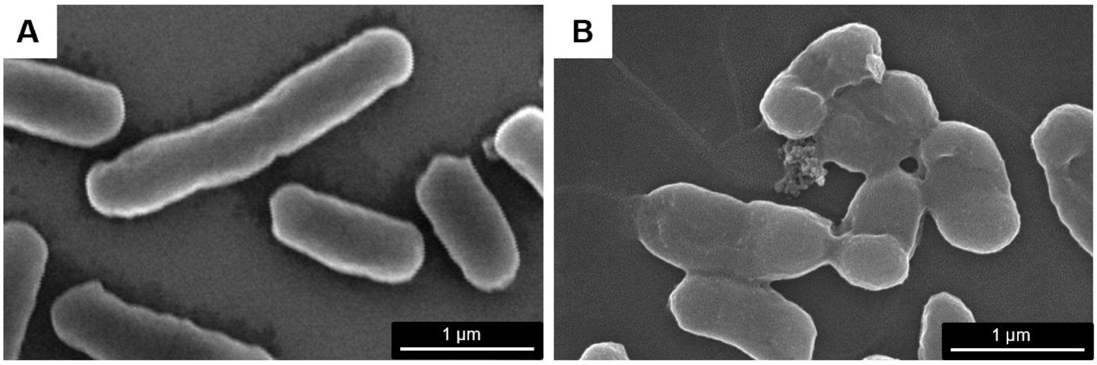

Figure S26. SEM images of *E. coli* (A) before and (B) after being treated with PDA/AgNCs-1.

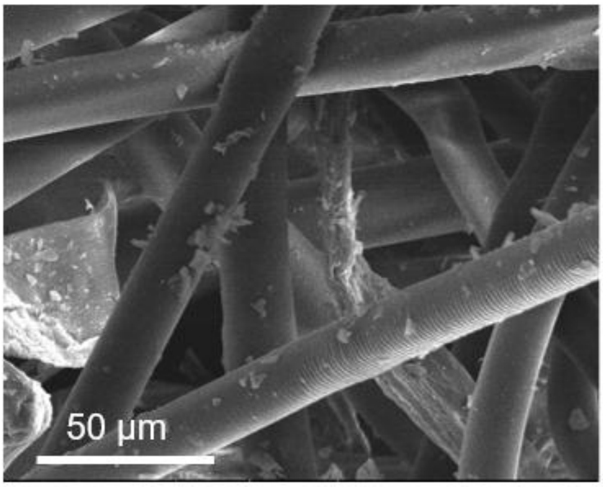

Figure S27. SEM image of the PDA/AgNCs/Cotton after 11-cycle tests.

Supplementary Tables

Table S1. The ligament sizes and the specific surface area of various silver aerogels reported in the literatures.

Aerogels	Precursors	Surface area ($m^2 \cdot g^{-1}$)	Ligament size (nm)	Refs.
Ag aerogel	AgNPs	5.2	21.9	Du et al.[1]
Ag nanowire aerogel	Silver nanowires	1	150	Zhu et al.[2]
Ag aerogel	Silver nanoshells	127	22.8	Gao et al.[3]
Ag aerogel	Silver nanoshells	81	43.7	Gao et al.[3]
Ag aerogel	Silver nanoshells	76	53.3	Gao et al.[3]
Ag nanowire aerogel	Silver nanowires	/	120	Huang et al.[4]
Ag aerogel	$AgNO_3$	2.1	48.2	Du et al.[5]
Ag aerogel	AgNPs	/	18.1	Du et al.[6]
Ag aerogel	$AgNO_3$	/	34.0	Yuan et al.[7]
Ag aerogel	AgNPs	15.0	/	Freytag et al.[8]
Ag aerogel	$AgNO_3$	10.0	/	Liu et al.[9]
Ag aerogel	$AgNO_3$	44.0	20	Panigrahy et al.[10]
Ag nanowires aerogel	Ag nanowires	5.5	113	Jung et al.[11]
Ag aerogel	$AgNO_3$	8.0	90	Georgi et al.[12]
**PDA/AgNCs**	**AgNCs**	**50.7**	**10.3**	**This work**

Table S2. Fitting results of MB in the presence of $NaBH_4$ and different catalysts with the pseudo-first-order kinetics.

Catalyst	k (min^{-1})	R^2
PDA/AgNCs-1	0.13	0.99
PDA/AgNCs-2	0.07	0.99
PDA/AgNCs-3	0.06	0.98
PDA	0.02	0.98
AgNCs	0.02	0.93

Table S3. Fitting results of MB in the presence of $NaBH_4$ and different doses of PDA/AgNCs-1 with the pseudo-first-order kinetics.

Dose of PDA/AgNCs (mg)	k (min^{-1})	R^2
0.2	0.03	0.96
0.4	0.13	0.99
0.8	1.19	0.98
1.6	4.30	0.95

Table S4. Comparison of catalytic results for the reduction of MB by $NaBH_4$ in the presence of various catalysts.

Catalysts	C_{MB} (mg mL^{-1})	C_{NaBH4} (mg mL^{-1})	$m_{Catalyst}$ (mg)	k (min^{-1})	k_{nor} (min^{-1} mg^{-1})	Refs.
3D-Graphene/Ag	0.314	1.110	5.0	0.25	0.05	Sahoo et al.[13]
Fe_3O_4@PS@Ag	0.005	0.080	2.0	0.53	0.27	Wang et al.[14]
Fe_3O_4@PPy-MAA/Ag	0.031	0.150	5.0	2.80	0.56	Das et al.[15]
Ag/Fe_3O_4@C	0.017	0.040	10.0	0.34	0.03	Zhu et al.[16]
Fe_3O_4@PDA-Ag	0.038	0.182	5.0	1.78	0.36	Cui et al.[17]
MOF@Ag	0.006	0.617	0.2	0.37	1.85	Lajevardi et al.[18]
Ag-TiO_2	0.020	0.378	0.3	0.56	1.87	Paramesh et al.[19]
Fe_3O_4@Ag/PDA	0.020	1.500	2.0	0.11	0.06	Zhang et al.[20]
PDA-kaolin-Ag	0.020	0.378	10.0	0.13	0.01	He et al.[21]
AgNPs/Thymbra	0.003	0.108	1.0	3.44	3.44	Veisi et al.[22]
Fe_3O_4@SiO_2-Ag	0.008	0.002	2.0	5.40	2.70	Mohammadi et al.[23]
**PDA/AgNCs**	**0.016**	**0.113**	**1.6**	**4.30**	**2.69**	**This work**

Table S5. Fitting results of MB in the presence of $NaBH_4$ and PDA/AgNCs-1 at different temperatures with the pseudo-first-order kinetics.

Reaction temperature (^{o}C)	k (min^{-1})	R^2
25	0.13	0.99
35	0.20	0.99
45	0.26	0.99

References

[1] R. Du, Y. Hu, R. Hübner, J.-O. Joswig, X. Fan, K. Schneider, A. Eychmüller, *Science Advances* **2019**, *5*, eaaw4590.

[2] W. Zhu, P. Wang, Z. Chen, C. Xu, Y. Jiao, M. Li, Y. Huang, *Journal of Materials Chemistry A* **2022**, *10*, 10780.

[3] X. Gao, R. J. Esteves, T. T. H. Luong, R. Jaini, I. U. Arachchige, *Journal of the American Chemical Society* **2014**, *136*, 7993.

[4] S. Huang, C. Feng, E. L. H. Mayes, B. Yao, Z. He, S. Asadi, T. Alan, J. Yang, *Nanoscale* **2020**, *12*, 19861.

[5] R. Du, J. Wang, Y. Wang, R. Hübner, X. Fan, I. Senkovska, Y. Hu, S. Kaskel, A. Eychmüller, *Nature Communications* **2020**, *11*, 1590.

[6] R. Du, J.-O. Joswig, X. Fan, R. Hübner, D. Spittel, Y. Hu, A. Eychmüller, *Matter* **2020**, *2*, 908.

[7] Y. Yuan, H. Zhao, W. Xv, D. Zhang, Z. Wang, H. Li, Y. Qin, S. Li, J. Lai, L. Wang, *Chinese Chemical Letters* **2022**, *33*, 2021.

[8] A. Freytag, S. Sánchez-Paradinas, S. Naskar, N. Wendt, M. Colombo, G. Pugliese, J. Poppe, C. Demirci, I. Kretschmer, D. W. Bahnemann, P. Behrens, N. C. Bigall, *Angewandte Chemie International Edition* **2016**, *55*, 1200.

[9] W. Liu, D. Haubold, B. Rutkowski, M. Oschatz, R. Hübner, M. Werheid, C. Ziegler, L. Sonntag, S. Liu, Z. Zheng, A.-K. Herrmann, D. Geiger, B. Terlan, T. Gemming, L. Borchardt, S. Kaskel, A. Czyrska-Filemonowicz, A. Eychmüller, *Chemistry of Materials* **2016**, *28*, 6477.

[10] S. Panigrahy, R. Mishra, P. Panda, M. Kempasiddaiah, S. Barman, *ACS Applied Nano Materials* **2022**, *5*, 8314.

[11] S. M. Jung, H. Y. Jung, M. S. Dresselhaus, Y. J. Jung, J. Kong, *Scientific Reports* **2012**, *2*, 849.

[12] M. Georgi, B. Klemmed, A. Benad, A. Eychmüller, *Materials Chemistry Frontiers* **2019**, *3*, 1586.

[13] P. K. Sahoo, N. Kumar, S. Thiyagarajan, D. Thakur, H. S. Panda, *ACS Sustainable Chemistry & Engineering* **2018**, *6*, 7475.

[14] Y. Wang, P. Gao, Y. Wei, Y. Jin, S. Sun, Z. Wang, Y. Jiang, *Journal of Environmental Management* **2021**, *278*, 111473.

[15] R. Das, V. S. Sypu, H. K. Paumo, M. Bhaumik, V. Maharaj, A. Maity, *Applied Catalysis B: Environmental* **2019**, *244*, 546.

[16] M. Zhu, C. Wang, D. Meng, G. Diao, *Journal of Materials Chemistry A* **2013**, *1*, 2118.

[17] K. Cui, B. Yan, Y. Xie, H. Qian, X. Wang, Q. Huang, Y. He, S. Jin, H. Zeng, *Journal of Hazardous Materials* **2018**, *350*, 66.

[18] A. Lajevardi, M. Tavakkoli Yaraki, A. Masjedi, A. Nouri, M. Hossaini Sadr, *Journal of Molecular Liquids* **2019**, *276*, 371.

[19] C. C. Paramesh, G. Halligudra, M. Muniyappa, M. Shetty, K. K. Somashekharappa, D. Rangappa, K. S. Rangappa, P. D. Shivaramu, *Ceramics International* **2021**, *47*, 14750.

[20] J. Zhang, R. Cao, W. Song, L. Liu, J. Li, *Journal of Colloid and Interface Science* **2022**, *607*, 1730.

[21] K. He, M. Yan, Z. Huang, G. Zeng, A. Chen, T. Huang, H. Li, X. Ren, G. Chen, *Chemosphere* **2019**, *219*, 400.

[22] H. Veisi, S. Azizi, P. Mohammadi, *Journal of Cleaner Production* **2018**, *170*, 1536.

[23] P. Mohammadi, H. Sheibani, *Applied Organometallic Chemistry* **2018**, *32*, e4249.
